# A spatial model of the plant circadian clock reveals design principles for coordinated timing

**DOI:** 10.15252/msb.202010140

**Published:** 2022-03-21

**Authors:** Mark Greenwood, Isao T Tokuda, James C W Locke

**Affiliations:** ^1^ Sainsbury Laboratory University of Cambridge Cambridge UK; ^2^ Department of Biochemistry University of Cambridge Cambridge UK; ^3^ Department of Mechanical Engineering Ritsumeikan University Kusatsu Japan; ^4^ Present address: Whitehead Institute for Biomedical Research Cambridge MA USA

**Keywords:** circadian clock, coordination, coupling, noise, plant, Plant Biology

## Abstract

Individual plant cells possess a genetic network, the circadian clock, that times internal processes to the day‐night cycle. Mathematical models of the clock are typically either “whole‐plant” that ignore tissue or cell type‐specific clock behavior, or “phase‐only” that do not include molecular components. To address the complex spatial coordination observed in experiments, here we implemented a clock network model on a template of a seedling. In our model, the sensitivity to light varies across the plant, and cells communicate their timing via local or long‐distance sharing of clock components, causing their rhythms to couple. We found that both varied light sensitivity and long‐distance coupling could generate period differences between organs, while local coupling was required to generate the spatial waves of clock gene expression observed experimentally. We then examined our model under noisy light‐dark cycles and found that local coupling minimized timing errors caused by the noise while allowing each plant region to maintain a different clock phase. Thus, local sensitivity to environmental inputs combined with local coupling enables flexible yet robust circadian timing.

## Graphical abstract

A spatial molecular model is developed to understand the design principles of plant clock coordination. The model shows that local cell‐to‐cell coupling combined with varied environmental signaling allows robust, yet flexible, timing.

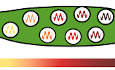

## Introduction

The circadian clock is a 24‐h genetic oscillator found in many organisms. The clock consists of a circuit of interlocking feedback loops of mRNAs and proteins that generate daily oscillations in circuit component levels. Signals from the environment align the timing of these oscillations to the day‐night cycle (Webb *et al*, 2019). Once set, circadian clocks act as an internal timing signal, allowing biological processes to anticipate the external environmental cycles. The clock modulates a diverse range of processes in plants, including cell division, tissue growth, flowering time, and scent emission (Nozue *et al*, 2007; Fenske *et al*, 2018; Fung‐Uceda *et al*, 2018; Greenwood & Locke, 2020). Altogether this daily timing provides a significant fitness advantage to the plant (Green *et al*, 2002; Dodd *et al*, 2005).

Individual plant cells possess a robust circadian clock (Gould *et al*, 2018). However, substantial differences in the period and phase of clock rhythms across the plant have been observed. Time‐lapse imaging experiments with luciferase and fluorescent reporter genes in the model plant *Arabidopsis thaliana* have shown that rhythms in core clock genes oscillate with different speeds in different organs under constant light (LL) (Thain *et al*, 2002; James *et al*, 2008; Yakir *et al*, 2011; Takahashi *et al*, 2015; Bordage *et al*, 2016; Gould *et al*, 2018). Further, experiments under a range of conditions have shown that differences in clock speed and phase can be caused by organs having different sensitivity to environmental signals (James *et al*, 2008; Bordage *et al*, 2016; Greenwood *et al*, 2019; Nimmo *et al*, 2020). Differences in the clock network between tissues may also contribute to generating differences in rhythms across the plant, as although the clock genes are broadly expressed (Bordage *et al*, 2016), some are tissue enriched (Endo *et al*, 2014), and mutations can affect organs differently (Takahashi *et al*, 2015; Lee & Seo, 2018; Nimmo *et al*, 2020).

The observed differences in clock rhythms across the plant raise the question of how clocks in different cells and tissues remain coordinated with each other. One mechanism would be for cells to communicate their timing with their neighbors, effectively coupling their rhythms. High‐resolution experiments have measured or inferred local coupling of clock rhythms between cells (Fukuda *et al*, 2007, 2012; Wenden *et al*, 2012; Endo *et al*, 2014; Takahashi *et al*, 2015; Gould *et al*, 2018; Greenwood *et al*, 2019), and local coupling can drive spatial waves of clock gene expression across the plant (Fukuda *et al*, 2007, 2012; Wenden *et al*, 2012; Gould *et al*, 2018; Greenwood *et al*, 2019). Longer distance coupling between clocks is also possible. For example, EARLY FLOWERING 4 (ELF4) communicates circadian temperature information over long distances by moving from the shoot to the root (Takahashi *et al*, 2015; Chen *et al*, 2020), and light information may be piped down the stem to entrain the root (Nimmo, 2018).

Mathematical modeling has played a crucial role in gaining a mechanistic understanding of plant circadian clocks. Models of the network have increased in complexity over time in parallel with the growing number of experiments (Locke *et al*, 2005a, 2005b, 2006; Zeilinger *et al*, 2006; Pokhilko *et al*, 2010, 2012; Fogelmark & Troein, 2014). Recently, these detailed molecular models have been used to probe the differences between the shoot and root clock (Bordage *et al*, 2016). However, the coupling of clocks between cells was not considered. For this, more computationally tractable models of the clock network are necessary. Reduced models of the network have already been constructed that capture many of the features of the single‐cell clock dynamics (Akman *et al*, 2012; De Caluwé *et al*, 2016; Foo *et al*, 2016; Tokuda *et al*, 2019). However, these models have not been applied to study spatial dynamics. Instead, “phase‐only” models that lack any genetic network information and only consider the phases of individual cellular rhythms have been preferred (Fukuda *et al*, 2007, 2012; Wenden *et al*, 2012; Gould *et al*, 2018). Although these models allow the simulation of general oscillatory behavior, owing to their simplicity they are unsuitable for investigating molecular mechanisms of the clock.

Recently, we used a “phase‐only” Kuramoto model (Kuramoto, 1975) to propose a mechanism for whole‐plant coordination of clocks (Gould *et al*, 2018; Greenwood *et al*, 2019). In order to match experimentally measured rhythms, we fixed clock periods to different speeds in each region of the plant. We assumed faster rhythms in the cotyledons, hypocotyl, and root tip, and slower rhythms in the rest of the root, as observed experimentally. With these periods fixed, cells were allowed to communicate clock phase through local cell‐to‐cell coupling. With these assumptions, simulations of the model generated waves of clock gene expression within and between organs, as observed experimentally (Gould *et al*, 2018; Greenwood *et al*, 2019). Thus, local cell‐to‐cell coupling could enable coordination between organs in plants.

Multiple questions remain about how the plant clock coordinates rhythms that cannot be addressed using a “phase‐only” model. For example, how are the periods set differently in different parts of the plant? What communication mechanisms allow the coupling of clock rhythms from cell‐to‐cell? How can the plant clock network “filter” both internal and environmental noise to robustly entrain to the environment? To begin to address these questions, in this work we developed a spatial network model of the plant circadian system. We modified a previously generated simplified network model and implemented it on a multicellular template of a seedling. In our model, the sensitivity to light varies across the plant, which can account for period differences between organs. We explore scenarios where cells communicate via local or long‐distance transport of clock components. Simulations with local coupling capture the spatial waves observed under LL, demonstrating a plausible mechanism of circadian coordination. In contrast, simulations with long‐distance coupling between the shoot and the root tip did not create spatial waves but could drive fast periods in the root tip. We applied our model with spatial differences in light sensitivity and local coupling to examine how plants keep time under noisy light‐dark (LD) cycles. We found that regional differences persist even under LD cycles, but cell‐to‐cell coupling minimized the error in timing caused by the noise. Thus, the combination of regional differences in sensitivity to inputs and local cell‐to‐cell coupling allows for coordinated timing in noisy environments.

## Results

### A locally coupled spatial model of the plant circadian clock network

**Figure 1:**
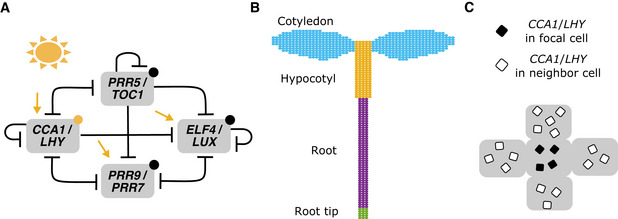
The structure of the spatial circadian clock model Summary of the modified compact circadian clock model used for simulations. The original compact model (De Caluwé *et al*, 2016) was modified to update the regulatory interactions and light inputs (Materials and Methods). Yellow arrows represent light‐induced synthesis, yellow circles light‐induced degradation, and black circles dark‐induced degradation. “T” arrows represent molecular repression.The network was implemented within each cell on a simplified template of a seedling, with cells classified as either cotyledon (blue), hypocotyl (yellow), root (purple), or root tip cells (green). Cells have a light sensitivity, *L_sens_*, that depends on the region (Materials and Methods).To simulate local coupling, the level of *CCA1*/*LHY* mRNA in the focal cell (black squares) was assumed to be coupled to the average level of the cell’s neighbors (white squares). The strength of the coupling is set by the *J_local_* parameter. We initially assumed coupling to occur through *CCA1*/*LHY* and also tested coupling through other components.

We first implemented a reduced network model of the *A. thaliana* circadian clock, hereafter referred to as the De Caluwé model (De Caluwé *et al*, 2016). To decrease the complexity of the model, the authors grouped functionally similar genes into single entities (Fig 1A). The compact network model incorporates known light inputs to the network including “acute” activation of *CIRCADIAN CLOCK ASSOCIATED 1* (*CCA1*)/*LATE ELONGATED HYPOCOTYL* (*LHY*) and *PSEUDO‐RESPONSE REGULATOR 9 (PRR9*)/*PRR7* at dawn, a constant increase in the synthesis of *CCA1*/*LHY* and *ELF4*/*LUX ARRHYTHMO* (*LUX*) under light, and altered degradation of several components in the light or dark (Fig 1A). The model qualitatively recapitulates clock dynamics under both LD cycles and LL, and at only 9 equations and 34 parameters is also computationally tractable for spatial simulations. We modified the De Caluwé model to include a repression rather than activation interaction between *CCA1*/*LHY* and *PRR9*/*PRR7* and included a term for *CCA1*/*LHY* repressing its own transcription, as these interactions have recently been shown experimentally (Adams *et al*, 2015). We also adjusted the degradation rate of PRR5/TOC1 to be higher in the dark, as several studies have established that the degradation of both proteins is increased in the dark (Más *et al*, 2003; Kiba *et al*, 2007; Kim *et al*, 2007; Fujiwara *et al*, 2008) (Appendix Table S1, Materials and Methods). The identified parameter values were robust to at least 5% variation (Appendix Fig S1, Materials and Methods).

We implemented the modified De Caluwé model on a simplified template of a seedling. The template consisted of approximately 800 cells, classified into cotyledon, hypocotyl, root, and root tip regions (Fig 1B, Materials and Methods). Although a number of studies have demonstrated local cell‐to‐cell coupling between clocks in *A. thaliana* (Fukuda *et al*, 2007, 2012; Wenden *et al*, 2012; Endo *et al*, 2014; Takahashi *et al*, 2015; Gould *et al*, 2018; Greenwood *et al*, 2019), the identity of the coupling signal, or signals, is unclear. Initially, to model the signal, the level of *CCA1*/*LHY* mRNA in one cell (Fig 1C, black squares) was assumed to be coupled to the average level of the cell’s neighbors (Fig 1C, white squares). The coupling strength, *J_local_*, determines the extent that molecules are shared (Materials and Methods). Although we did not model diffusion directly, this coupling function was designed to simulate the passive movement of molecules which commonly occurs through plasmodesmata in plants (Faulkner, 2018). To simulate period variation between cells, at each time step, we multiplied the level of the mRNA and protein by a “scaling” parameter. For each cell, this parameter was randomly selected from a normal distribution to give a unique value for each cell through the simulation (Materials and Methods). This approach generates between‐cell but not within‐cell period differences, allowing us to focus on the one source of variation. Further, we could set the range of the distribution differently for each organ, as informed by experimental data (Appendix Fig S2).

### Different light sensitivities can explain organ‐level differences in phase and period

**Figure 2:**
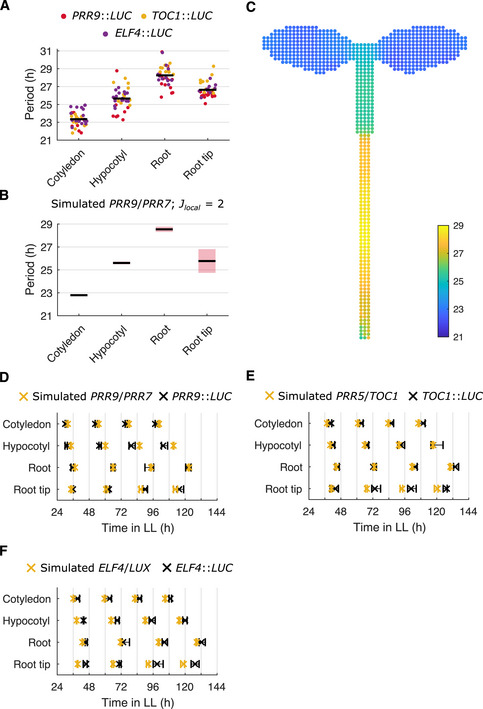
Regional differences in light sensitivity can generate the period structure observed experimentally APeriod estimates of *PRR9*::*LUC*, *TOC1*::*LUC*, and *ELF4*::*LUC* for different organs imaged under LL. Each data point represents a period estimate from the organ of a single seedling. The horizontal black line shows the mean.B, CPeriod estimates of simulated *PRR9*/*PRR7* expression, measured from regions within the seedling template (B) or individual cells of the seedling template (C). In (B), the black line indicates the mean and the red shaded area one SD of 9 independent simulations. In (C), the color of the cell represents the periods of the individual oscillations. By assuming higher light sensitivity of cells in the cotyledon, hypocotyl, and root tip, the model approximates the period differences observed between regions in experiments. A noise parameter was set to a different value for each region, as informed by single‐cell experiments (Appendix Fig S2). Simulations assumed local cell‐to‐cell coupling (*J_local_* = 2).D–FTimes of the final peaks of simulated *PRR9*/*PRR7* and *PRR9*::*LUC* (D), simulated *PRR5*/*TOC1* and *TOC1*::*LUC* (E), or simulated *ELF4*/*LUX* and *ELF4*::*LUC* (F), in different organs measured under LL. Simulations assumed varied light sensitivities and local cell‐to‐cell coupling (*J_local_* = 2). Data points represent the 25‐th percentile, median, and the 75‐th percentile for the peak times of oscillations scored as rhythmic, *n* = 9 simulations. Data information: Experimental data is an analysis of *Arabidopsis* time‐lapse movies carried out previously (Greenwood *et al*, 2019). For *PRR9*::*LUC* data *N* = 4; *TOC1*::*LUC* data *N* = 3; *ELF4*::*LUC* data *N* = 3. For all, *n* = 7–18. *N* represents the number of independent experiments and *n* the total number of organs tracked.

We next attempted to recapitulate in our model the differences in clock period and phase in different organs that have been observed in experiments using a single‐cell CCA1‐YFP reporter (Gould *et al*, 2018) and a *GIGANTEA* luciferase reporter (Greenwood *et al*, 2019). In these experiments, faster rhythms were observed in the cotyledon, hypocotyl, and root tip, with slower rhythms in the rest of the root. We first reanalyzed existing luciferase data (Greenwood *et al*, 2019) and confirmed that these relationships held for several of the core clock genes in our model, *PSEUDO‐RESPONSE REGULATOR 9* (*PRR9)*, *TIMING OF CAB EXPRESSION 1* (*TOC1*), and *ELF4* (Fig 2A). Whereas in our previous “phase‐only” model, we fixed the periods to be different in each part of the plant, with our spatial network model, we could now investigate what causes the differences in periods. Previously it has been hypothesized that varied sensitivities to light alter periods across the plant (Bordage *et al*, 2016; Greenwood *et al*, 2019; Nimmo *et al*, 2020). This was based on previous experiments demonstrating: (i) period differences between organs when exposed to equal amounts of light (Bordage *et al*, 2016; Greenwood *et al*, 2019); (ii) the loss of some period differences in the light‐sensing mutant *phyb‐9* (Greenwood *et al*, 2019; Nimmo *et al*, 2020); and (iii) the spatial expression pattern of *PHYB* and other light‐sensing genes (Somers & Quail, 1995; Bognár *et al*, 1999; Tóth *et al*, 2001). Experiments, however, are confounded by changes in metabolism and development caused by light (Nozue *et al*, 2011). We therefore tested the light‐sensitivity hypothesis in our spatial model, by setting the sensitivity to light to differ depending on the region, and examining whether this could generate the period differences observed across the plant.

**Figure EV1:**
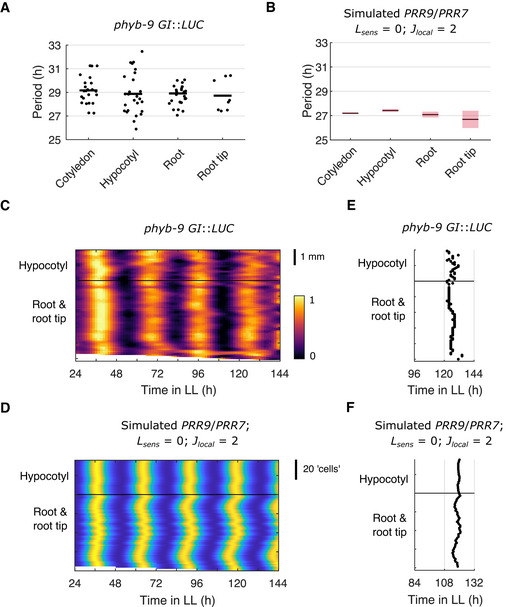
Simulations with zero light input capture the loss of period differences observed in the *phyb‐9* mutant APeriod estimates of *GI*::*LUC* expression measured from different organs under red light in the light‐sensing mutant *phyb‐9* genetic background. Each data point represents a period estimate from the organ of a single seedling and the horizontal black line shows the mean.BPeriod estimates of simulated *PRR9*/*PRR7* expression, measured from regions within the template seedling. Simulations were performed with zero light sensitivity for all cells (*L_sens_* = 0; *D* = 1) and with local cell‐to‐cell coupling (*J_local_* = 2). The horizontal black line shows the mean and the red shaded area one SD of 9 simulations.CRepresentative intensity plot of *GI*::*LUC* expression across longitudinal sections of a single seedling under constant red light. Imaging was performed under red light in the light‐sensing mutant *phyb‐9* genetic background.DRepresentative intensity plot of simulated *PRR9*/*PRR7* expression across longitudinal sections of a single seedling under LL. Simulations were performed with zero light sensitivity for all cells (*L_sens_* = 0; *D* = 1) and with local cell‐to‐cell coupling (*J_local_* = 2).E, FTimes of the final peaks of *GI*::*LUC* intensity plot (E) or simulated *PRR9*/*PRR7* expression (F). Simulations were performed with zero light sensitivity for all cells (*L_sens_* = 0; *D* = 1) and with local cell‐to‐cell coupling (*J_local_* = 2). Data information: Experimental data is an analysis of *Arabidopsis* time‐lapse movies carried out previously (Greenwood *et al*, 2019). *N* = 2 and *n* = 8–23, where *N* represents the number of independent experiments and *n* the total number of organs tracked.

We entrained the cells in our simulations to LD cycles for 4 days before releasing them into LL for a further 6 days and measured the periods, as carried out in previous experiments (Greenwood *et al*, 2019). We initially set the local cell‐to‐cell coupling parameter, *J_local_*, to 2. When assuming high sensitivity to light in the cotyledon (*L_sens_* = 1.6) and hypocotyl (*L_sens_* = 1.0), but lower in the root (*L_sens_* = 0.65) and root tip (*L_sens_* = 0.95), all regions entrained to the LD cycles (Appendix Fig S3). Upon transfer to LL, we were able to generate different periods (Fig 2B and C) and phases (Fig 2D–F and Appendix Fig S4) in each organ, matching those observed experimentally. This is due to higher light sensitivity causing the clock to run faster in our simulations (Appendix Fig S5), as expected for a diurnal organism (Aschoff & Pohl, 1978). Finally, by setting the sensitivity to light in our locally coupled model to be zero in all regions of the seedling, we were also able to simulate the loss of period differences observed in the light‐sensing mutant *phyb‐9* (Greenwood *et al*, 2019) (Fig EV1A and B). Thus, our results revealed that different sensitivities to environmental inputs are sufficient to generate the experimentally observed spatial differences in period and phase across the plant.

### Local sharing of clock components can drive spatial waves of clock gene expression

**Figure 3:**
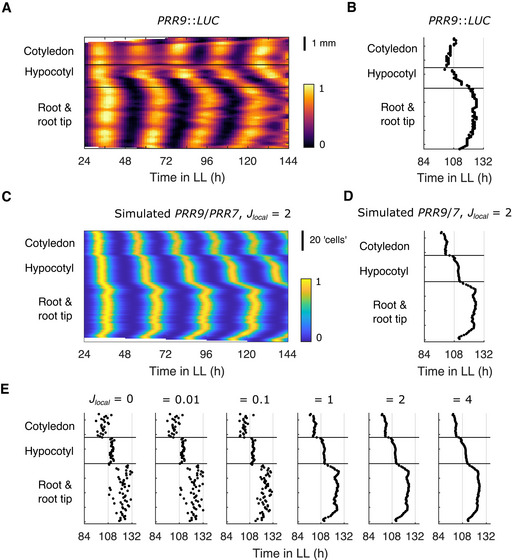
Local sharing of clock components can reproduce experimentally observed spatial waves of clock gene expression Representative intensity plot of *PRR9*::*LUC* expression measured from longitudinal sections of a single seedling under LL.Times of the final peaks of the *PRR9*::*LUC* intensity plot.Representative intensity plot of simulated *PRR9*/*PRR7* expression measured from longitudinal sections of a single seedling under LL. Simulations assumed varied light sensitivities and local cell‐to‐cell coupling (*J_local_* = 2).Times of the final peaks of the simulated *PRR9*/*PRR7* intensity plot.Times of the final peaks of simulated *PRR9*/*PRR7* intensity plots, each simulated under LL with increasing strengths of local cell‐to‐cell coupling. Data information: Experimental data is an analysis of *Arabidopsis* time‐lapse movies carried out previously (Greenwood *et al*, 2019). *N* = 4 and *n* = 7–14, where *N* represents the number of independent experiments and *n* the total number of organs tracked. Data in (D) is replotted within (E) as “*J_local_* = 2” and Fig EV2A as “*CCA1*/*LHY* coupled”. (E) is replotted as Fig EV3A and Appendix Fig S8A.

Previously we observed two waves of clock gene expression, one traveling up, and one down the root, in *CCA1*, *PRR9*, and *GI* reporters (Gould *et al*, 2018; Greenwood *et al*, 2019). These waves could be explained in a “phase‐only” model by local cell‐to‐cell coupling (Fukuda *et al*, 2007, 2012; Gould *et al*, 2018; Greenwood *et al*, 2019). We next tested whether a plausible mechanism for cell‐to‐cell coupling, sharing of mRNA between cells (Maizel *et al*, 2020), can recapitulate the experimental observations. To provide a benchmark, we first analyzed the seedlings carrying transcriptional reporters for *PRR9*, *TOC1*, and *ELF4* (Greenwood *et al*, 2019) at the sub‐tissue level (Materials and Methods). As in previous studies (Fukuda *et al*, 2007, 2012; Wenden *et al*, 2012; Gould *et al*, 2018; Greenwood *et al*, 2019), space‐time plots revealed spatial waves of gene expression within and between organs (Fig 3A, Appendix Fig S6A and C and Movie EV1). The direction of the waves could be clearly observed in plots of the final peaks of expression (Fig 3B and Appendix Fig S6E and G). For each gene, the wave directions appeared similar, traveling from the faster oscillating regions to the slower oscillating regions. Particularly distinct were the waves converging from the hypocotyl and root tip to the slower oscillating root region.

**Figure EV2:**
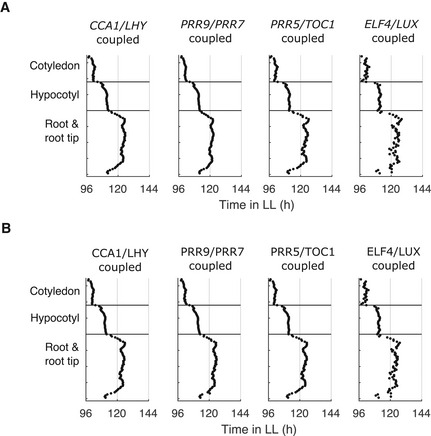
Peaks of simulated expression with the local sharing of different clock molecules between cells A, BThe times of the final peaks of simulated *PRR9*/*PRR7* intensity plots, simulated with the sharing of *CCA1*/*LHY*, *PRR9*/*PRR7*, *PRR5*/*TOC1*, or *ELF4*/*LUX* mRNA (A) or protein (B) locally between neighbor cells. Simulations were performed under LL with local cell‐to‐cell coupling (*J_local_* = *2)*. Data information: Data in (A) (“*CCA1*/*LHY* coupled”) is replotted from Fig 3D and data in (B) (“ELF4/LUX coupled”) is replotted from Fig 4B.

**Figure EV3:**
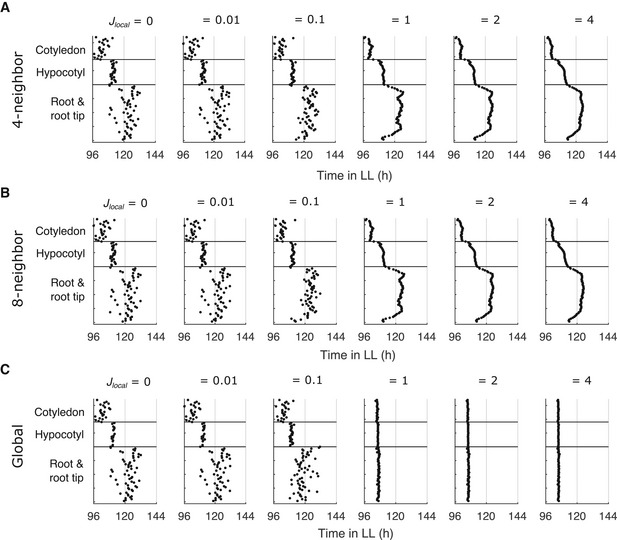
Peaks of simulated expression under LL with different coupling rules A–CThe times of the final peaks of simulated *PRR9*/*PRR7* intensity plots, simulated under LL with increasing strengths of local cell‐to‐cell coupling between the 4 nearest neighbor cells (A), 8 nearest neighbor cells (B), or globally (all‐to‐all; C). Data information: (A) is replotted from Fig 3E.

We next analyzed at the sub‐tissue level the simulations performed with varied light sensitivity and local coupling (Appendix Fig S7B, Materials and Methods), to see if they capture the spatial dynamics. For each gene, the wave patterns appeared similar to experiments, traveling from the fast oscillating regions that are more sensitive to light, into the slower regions with lower sensitivity to light (Fig 3C and D, Appendix Fig S6B, D, F and H, and Movie EV2). These waves required cell‐to‐cell coupling through the local sharing of clock components, as we only observed waves with coupling strengths, *J_local_*, above approximately 1 (Fig 3E and Appendix Fig S8). Similar simulation results were found when keeping regional differences of light sensitivity and local cell‐to‐cell coupling, but assuming no cell‐to‐cell variability within regions (Appendix Fig S9). We also found that our simulation results were qualitatively similar when using different clock gene mRNA (Fig EV2A) or protein (Fig EV2B) as the coupling component, suggesting that any cell‐to‐cell sharing of clock components could explain the experimentally observed spatial dynamics. Additionally, we ran simulations assuming local coupling between 8 rather than 4 neighbor cells, and simulations assuming global (all‐to‐all) coupling. Increasing the local coupling to be between 8 neighbor cells gave similar results (Fig EV3A and B). However, with global coupling all cells adopted the same phase at higher coupling strengths, regardless of position in the plant (Fig EV3C). Finally, we further analyzed our simulations in which we set the sensitivity to light input to be equal in all regions of the seedling. At the sub‐tissue level, we observed the loss of spatial waves (Fig EV1D and F) seen in the light‐sensing mutant *phyb‐9* (Greenwood *et al*, 2019) (Fig EV1C and E). Taken together, these results show that the assumptions of local cell‐to‐cell coupling and differential light sensitivity between regions are the key aspects of our model that allow a match to experimental data.

### Long‐distance sharing of clock components can generate period differences

**Figure 4:**
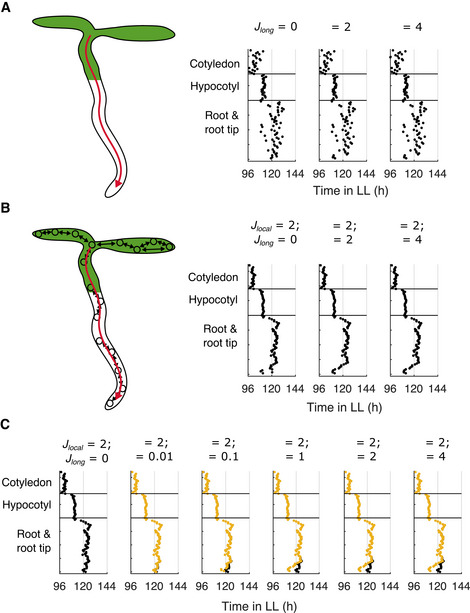
Long‐distance sharing of clock components does not generate spatial waves but does generate period differences between organs A, BTimes of the final peaks of the simulated *PRR9*/*PRR7* intensity plot simulated under LL. Simulations were performed with increasing strengths of long‐distance coupling, *J_long_,* without (A) or with (B) local cell‐to‐cell coupling (*J_local_* = *2*). Both long and local coupling acts through the sharing of ELF4/LUX. The schematics represent the long‐distance (red arrow) and local (black arrows) coupling present in the adjacent simulations.CTimes of the final peaks of simulated *PRR9*/*PRR7* intensity plot under LL, without the assumption of higher light sensitivity in the root tip. Simulations were performed with local cell‐to‐cell coupling (*J_local_* = *2*) and without (black dots) or with (yellow dots) increasing strengths of long‐distance coupling. Data information: Data in (B) (“*J_local_* = *2*; *J_long_* = *0”*) is replotted within Fig EV2B as “ELF4/LUX coupled”.

In addition to local coupling, the long‐distance shoot‐to‐root movement of ELF4 has been proposed to couple clocks in different organs (Chen *et al*, 2020). We simulated seedlings assuming that ELF4 protein expressed from cells in the shoot is shared with cells in the root tip region. We did this to approximate a known destination for phloem signals in plants (Oparka *et al*, 1994), and the location that ELF4 is observed in experiments (Chen *et al*, 2020). In these simulations, the peak times of cells in the root tip became closer to those in the shoot, but we did not see the spatial waves of gene expression observed in experiments (Fig 4A). We then simulated seedlings with combinations of local and long‐distance coupling, to see if together they improved the match to experimental data. In these simulations, we saw a clear spatial structure with waves of clock gene expression (Fig 4B), matching previous simulations with only local coupling (Fig 3). These simulations confirm that it is the local, not long‐distance, coupling that drives the spatial waves of gene expression.

We hypothesized that long‐distance coupling instead contributes by matching clock periods between different organs of the plant, as has been proposed for ELF4 (Chen *et al*, 2020) and light piping from the shoot to the root (Nimmo, 2018). To test this in our model, we removed the assumption of high light sensitivity at the root tip (Materials and Methods), and re‐simulated the model with combinations of local and long‐distance coupling. Without long‐distance coupling (*J_long_* = 0), as expected, cells were slower at the root tip, and the spatial wave up the root was lost (Fig 4C, black). However, with *J_long_* above approximately 0.1, we observed an increase in the speed of cells at the root tip, and the recovery of spatial waves up the root (Fig 4C, yellow). This approximated the typical spatial structure observed with higher light sensitivity at the root tip (Fig 4B). Together, this suggests that although long‐distance coupling does not generate spatial waves, it can create period differences between regions of the plant. Further experiments will be required to understand the extent of long‐distance clock coupling during plant development, so henceforth we focus on our model assuming different sensitivities to light and only local coupling.

### Local flexibility persists under LD cycles

**Figure 5:**
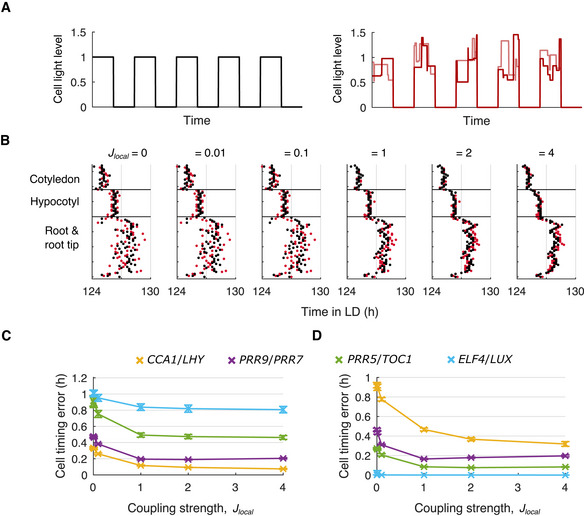
Local coupling reduces cell timing errors under noisy LD cycles ASchematic of idealized (left) and noisy (right) LD cycles, without or with fluctuations in light levels during the daytime, respectively. Each line represents the LD cycle for a single cell.BTimes of the final peaks of simulated *PRR9*/*PRR7* intensity plots, simulated under idealized (black dots) or noisy (red dots) LD cycles with increasing strength of local cell‐to‐cell coupling.C, DThe cell timing error was calculated from the peaks (C) or troughs (D) of simulated gene expression, with increasing strength of local cell‐to‐cell coupling. Data points represent the mean ± SD, *n* = 9 simulations. Data information: Data in (B) (“*J_local_* = *2”*) is replotted within Appendix Fig S11B as “12‐h day”. (B) is replotted as Appendix Fig S10B. (C) and (D) are replotted as Appendix Fig S13A.

**Figure EV4:**
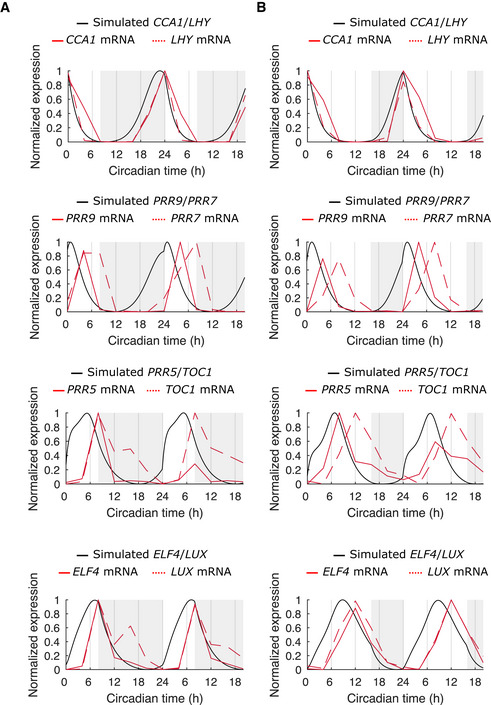
Simulations under short‐ and long‐day lengths approximate experimental data A, BSimulated (black line) and experimental (red line) expression of clock genes under idealized LD cycles with 8 h (A) or 16 h (B) day lengths. Simulations were implemented as a single cell, without variation in gene expression, and with *L_sens_* = 1. Data information: Experimental data are *Arabidopsis* whole‐plant averages obtained from the DIURNAL database (Mockler *et al*, 2007).

We simulated our locally coupled model, with the sharing of *CCA1/LHY* mRNA, under LD cycles to investigate whether local flexibility persisted with rhythmic input. We first simulated idealized LD cycles, where light is fully on during the daytime and off at night (Fig 5A, left; Materials and Methods). The sensitivity of cells to light during the daytime varied between regions, as with LL simulations, and we included local cell‐to‐cell coupling. Under LD cycles, cells had more similar timings than under LL; however, inspection of the peaks of *PRR9*/*PRR7* expression revealed differences between regions (Fig 5B, black dots). These phase differences were qualitatively the same as observed under LL (Fig 3) and persisted even at high coupling strengths (Fig 5B, black dots). Also as observed under LL, with *J_local_* above approximately 1, spatial waves could be observed (Fig 5B, black dots). These simulations qualitatively match the experimentally observed spatial waves under LD cycles (Greenwood *et al*, 2019). A similar structure was observed in the other clock genes (Appendix Fig S10, black dots), although spatial waves of *PRR5*/*TOC1* and *ELF4*/*LUX* were altered because their peaks coincided with the LD transition which had a synchronizing effect (Appendix Fig S10C and D, black dots). Simulations also captured whole‐plant experimental data under short or long days (Fig EV4) and predicted that a spatial structure remains under these conditions (Appendix Fig S11).

### Cell‐to‐cell coupling improves timing under noisy LD cycles

Our modeling suggested that different sensitivities to environmental inputs across the plant allow the clock to be locally flexible, by allowing it to adopt different phases across the plant even under LD cycles. However, it was not clear whether a locally flexible clock would still entrain to environmental cycles and maintain a stable spatial structure of phase differences when the environment is noisy, as is the case outside of the laboratory. To test this, we simulated more realistic LD cycles containing fluctuations in the light intensity (Fig 5A, right). We first considered the scenario where the fluctuations in light intensity are uncorrelated for neighboring cells, to simulate microenvironmental differences (Materials and Methods). Without cell‐to‐cell coupling (*J_local_* = 0) the peak times of *PRR9*/*PRR7* expression were highly variable within a region and any spatial structure was difficult to resolve (Fig 5B, red dots). However, with increasing strengths of coupling we observed a decrease in variability, revealing a spatial structure (Fig 5B, red dots) similar to that observed under idealized LD cycles (Fig 5B, black dots). A similar effect was observed for the remaining clock genes (Appendix Fig S10, red dots). Overall, local cell‐to‐cell coupling appears to reduce the within‐region variability of cellular rhythms. Yet, despite the stabilizing effect of the coupling, between‐region differences persist, due to the between‐region differences in sensitivity to the environment.

This emergence of spatial structure suggests that local coupling improves the timing of individual cells under noisy environments. To quantify this effect, we measured the timing of the peaks (Fig 5C and Appendix Fig S12) and troughs (Fig 5D and Appendix Fig S12) of expression relative to simulations without noise in the LD cycle (Materials and Methods). The timing of each gene was mis‐timed under noisy LD cycles compared to idealized LD cycles. For example, with *J_local_* = 0, there was a timing error of 0.48 ± 0.02 h (mean ± SD) for the peaks, and 0.46 ± 0.02 h for the troughs of *PRR9*/*PRR7* expression. Each gene showed a differing degree of error in their peak (Fig 5C and Appendix Fig S12) and trough (Fig 5D and Appendix Fig S12) times, which reflects differences in their regulation under light and dark conditions (Appendix Fig S12 and Appendix Table S1). For example, the troughs of *ELF4*/*LUX* expression were synchronized (Fig 5D, blue line) due to rapid degradation in the dark (Appendix Fig S12D). However, for each gene, we observed a decrease in the timing error with increasing strengths of local cell‐to‐cell coupling (Fig 5C and D). This is consistent with local coupling having a stabilizing effect on cellular oscillations, increasing their robustness to perturbation from the noisy environment. Local cell‐to‐cell coupling similarly reduced the timing error when we assumed weak correlations in the LD cycles that cells are exposed to, to simulate shared microenvironments (Appendix Fig S13A–C, Materials and Methods). However, local coupling no longer reduced the timing error when we assumed stronger correlations in the LD cycles, as neighboring cells experienced more similar LD cycles (Appendix Fig S13D and E). Taken together, our results show that by averaging fluctuations between neighboring cells, local cell‐to‐cell coupling can improve timing accuracy of individual cells under noisy environments. Because coupling acts locally, between‐region differences in phase, due to between‐region differences in light sensitivities, are able to persist despite the averaging effect of cell‐to‐cell coupling.

## Discussion

Here, we developed a spatial network model for the plant clock and used it to examine the design principles of clock coordination in plants. We found that regional differences in sensitivity to light and local cell‐to‐cell coupling through the sharing of clock components can capture the period differences and phase waves across the plant observed in previous time‐lapse experiments. In addition, we found that long‐distance coupling can generate period differences between regions but cannot on its own generate spatial waves. Our simulations found that local coupling combined with regional differences in sensitivity to light allows phase flexibility between regions to persist under noisy LD cycles while minimizing timing errors. We therefore found that the plant circadian clock system can combine regional differences in environmental signaling with local cell‐to‐cell coupling to enable robust, yet flexible, circadian timing under noisy environmental cycles.

In this work, we found that regional differences in timing can be explained by varied sensitivities to the environment. The assumption of regional differences in sensitivity is based on previous experiments demonstrating that period differences across the plant are dependent on light input through PHYB (Greenwood *et al*, 2019; Nimmo *et al*, 2020). For example, in WT seedlings, the root tip oscillates faster than the root, but this difference is lost in the *phyb‐9* mutant (Greenwood *et al*, 2019). We represent this in the model by assuming the root tip is more sensitive to light than the rest of the root. Also, mutations in the evening complex components, *ELF3, ELF4, and LUX,* have different effects on the clock in roots compared to shoots (Nimmo *et al*, 2020), suggesting organ‐specific functions, and the evening complex is known to interact with PHYB (Huang & Nusinow, 2016), suggesting it may mediate between‐organ differences in environmental inputs.

This mechanism of local differences in environmental sensitivity could allow regions to adapt their entrainment to local environmental conditions. Another mechanism that can generate differences in timing is that biochemical parameters, such as degradation rates, can vary between regions of the plant (Vanselow *et al*, 2006; Relógio *et al*, 2011; Yoo *et al*, 2013). In the future, it will be interesting to compare these two different mechanisms of generating differences in clock timing. One possibility is that regional differences in sensitivity to the environment allow better entrainment to the complex and changing environments found in the natural environment. For example, temperature is an entraining cue for the clock that is phase shifted between the air and soil, with the size of the phase shift varying seasonally (Dawson & Fisher, 1964). Although technically challenging, experiments under more complex environments, with differences in conditions between the shoot and the root, will be important for testing this hypothesis. Promising advances in this direction include the GLO‐Roots system (Rellán‐Álvarez *et al*, 2015), and the robotics of Bordage *et al* (2016), which allow the root to be maintained in a different environment.

We investigated two different mechanisms contributing to clock coordination across the plant: local cell‐to‐cell coupling and long‐distance transport. Our simulations, as well as previous experiments (Fukuda *et al*, 2007, 2012; Gould *et al*, 2018; Greenwood *et al*, 2019), suggest that local cell‐to‐cell coupling can coordinate rhythms across *Arabidopsis* seedlings. Local cell‐to‐cell coupling has also been proposed to coordinate rhythms in other plant systems, including *Lemna gibba* fronds (Muranaka & Oyama, 2016; Ueno *et al*, 2021). The long‐distance movement of clock proteins and light piped from the shoot has also been proposed to coordinate rhythms (Nimmo, 2018; Chen *et al*, 2020). In our long‐distance simulations presented here, we assumed ELF4 levels in the root tip are coupled to ELF4 levels in the hypocotyl. We based this assumption on the work of Chen *et al*, 2020, which showed that when shoots overexpressing an ELF4‐GFP construct are grafted to *elf4* roots, ELF4‐GFP is observed in cells close to the phloem unloading zone at the root tip (Chen *et al*, 2020). Our simulations suggest that this long‐distance transport could contribute to the period structure within a plant but alone cannot generate the spatial waves of gene expression observed in the experiment. However, we did not consider secondary unloading sites of long‐distance transport (De Schepper *et al*, 2013). Further, we did not consider the time lag due to the transport of molecules (Knox *et al*, 2018), which may destabilize rhythms. In future work it will be important to monitor rhythms within *Arabidopsis* plants throughout their development, in order to understand the contribution of different mechanisms to whole‐organism coordination.

The molecular agents driving the coordination of the plant circadian system are still being deciphered. As discussed above, ELF4 protein and light piping have been proposed to mediate long‐distance coupling (Nimmo, 2018; Chen *et al*, 2020). Although the signals mediating local cell‐to‐cell coupling are unclear, it is known that long‐distance signals must also move locally in order to reach the translocation stream (Faulkner, 2018). This raises the possibility that long‐distance coupling signals additionally couple cells locally. Further, as explored in our simulations, it is possible that other clock components also act as local coupling signals. Of particular interest will be *TOC1* and *GI*, as grafting combined with transcriptomics has shown that their mRNA can move between the shoot and the root (Thieme *et al*, 2015), indicating they could be candidates as local and/or long‐distance coupling signals.

In the future, it will also be important to increase the realism of the model. For example, in our simulations, we focused on between‐cell variations in timing using a “scaling parameter” approach, which scales the mRNA and protein levels in each cell in order to generate cell‐to‐cell variability in periods (Materials and Methods). This approach is computationally simple while allowing us to set different levels of between‐cell period variability in each organ, as observed experimentally, but it does not explicitly model the noise in the molecular reactions. Modeling molecular noise explicitly by means of stochastic equations has previously revealed unexpected properties of circadian clocks, such as robustness to low molecule numbers (Gonze *et al*, 2002; Forger & Peskin, 2005; Zhang & Gonze, 2021) and improved re‐entrainment (Guerriero *et al*, 2014). Such an approach applied to our spatial model would allow an in‐depth investigation of both between and within cell period variation, and may reveal further roles for intercellular coupling.

Previous models of the plant circadian clock have proven important for understanding aspects of plant physiology, including starch metabolism and flowering (Seaton *et al*, 2014, 2015). However, it was recently shown that the clock controls physiology in a tissue‐specific manner. For example, clocks in the epidermis regulate growth, whereas those in the vasculature regulate flowering (Shimizu *et al*, 2015). In future, our spatial model could be extended to investigate circadian control of physiology at the cell and tissue level. An interesting example will be cell division, which is regulated by the clock (Fung‐Uceda *et al*, 2018), and occurs in meristematic regions of the plant (Meyerowitz, 1997). It will be interesting to see if, and how, flexibility of the circadian system impacts tightly controlled phenotypes such as this.

The plant circadian system, with local inputs to cells that are coupled together, represents a decentralized structure. This is in contrast to the centralized mammalian circadian system, where the suprachiasmatic nucleus in the brain receives the key entraining signal of light and then couples rhythms across the body (Bell‐Pedersen *et al*, 2005). Here we found that this decentralized structure can afford plants flexibility, filtering noise but allowing regions to adopt different phases.

## Materials and Methods

### Single‐cell molecular model

As a model for the plant circadian clock, we utilized the compact model introduced by De Caluwé *et al* (2016). The original compact model consists of 9 ordinary differential equations. Among them, 8 equations describe the temporal evolution of the mRNA and protein levels of the core clock genes. The clock genes are grouped into four sets of pairs labeled as: CL (*CCA1* and *LHY*), P97 (*PRR9* and *PRR7*), P51 (*PRR5* and *TOC1*), and EL (*ELF4* and *LUX*). The 9‐th equation is for protein P, a yet‐identified protein which mediates the acute activation of transcription by light observed in experiments (Ito *et al*, 2005; Locke *et al*, 2005b).

In our modeling, we modified the original De Caluwé model. According to the experiments reported in Para *et al* (2007), the compact model assumed that the P97 variable is activated by CL. However, more recent work has shown that *LHY* acts as a repressor of *PRR9* and *PRR7* (Fogelmark & Troein, 2014; Adams *et al*, 2015). We therefore replaced the activation term that represents the connection from CL to P97 with a repression. In addition, we added a term for CL repressing its own transcription, as this has also recently been observed for *CCA1* and *LHY* (Adams *et al*, 2015).

We also made modifications to the light input mechanism. In the original De Caluwé model, light signals feed into the network at multiple levels: (i) The transcription of CL and P97 is acutely activated at dawn by the dark‐accumulating protein P; (ii) The transcription of EL is increased continuously by light; (iii) The translation of CL is increased by light; (iv) The degradation rate of CL mRNA is increased by light; (v) The degradation rate of P51 protein is increased in the light; and (vi) The degradation rates of P97 and EL proteins are increased in the dark. In our model, we adjusted the degradation rate of P51, as several studies have established that the degradation of both *PRR5* and *TOC1* are also increased in the dark (Más *et al*, 2003; Kiba *et al*, 2007; Kim *et al*, 2007; Fujiwara *et al*, 2008) (Appendix Table S1).

Additionally, we introduced *L_sens_*, to represent the cellular sensitivity to light. In the presence of light *L* = 1, a cell senses light according to its sensitivity, *L_sens_ L* > 0. In the absence of light *L* = 0, the cell does not sense light, *L_sens_ L* = 0. We include *L_sens_* in the terms describing protein P degradation, EL and CL translation, and CL mRNA degradation, as in these terms light increases the rates of reaction. We additionally include *L_sens_* in the term describing the degradation of the EC, as it has previously been suggested that ELF3 degradation (an EC component not explicitly modeled in De Caluwé) is increased in the light (Pokhilko *et al*, 2012). *L_sens_* was set to 1 in the single cell implementation of the model, but varied between cells in the multicellular implementation (see “Spatial molecular model”). The revised single‐cell model is now described by the following differential equations:

$$\frac{dc_{\mathrm{CL}}^{\left(m\right)}}{\mathrm{dt}}=\left(v_{1\;}+v_{1L}L_{\mathrm{sens}}\;L\left(t\right)P\right)\frac{1}{1+{\left(\frac{c_{\mathrm{CL}}^{\left(p\right)}}{K_{0}}\right)}^{2}+{\left(\frac{c_{P97}^{\left(p\right)}}{K_{1}}\right)}^{2}+{\left(\frac{c_{P51}^{\left(p\right)}}{K_{2}}\right)}^{2}}\;‐\left(k_{1L}L_{\mathrm{sens}}\;L\left(t\right)+k_{1D}D\left(t\right)\right)c_{\mathrm{CL}}^{\left(m\right)},$$

$$\frac{dc_{\mathrm{CL}}^{\left(p\right)}}{\mathrm{dt}}=\left(p_{1}+p_{1L}L_{\mathrm{sens}}\;L\left(t\right)\right)c_{\mathrm{CL}}^{\left(m\right)}‐d_{1}c_{\mathrm{CL}}^{\left(p\right)},$$

$$\frac{dc_{P97}^{\left(m\right)}}{\mathrm{dt}}=\left(v_{2L}L_{\mathrm{sens}}\;L\left(t\right)P+v_{2A}\right)\frac{1}{1+{\left(\frac{c_{P51}^{\left(p\right)}}{K_{4}}\right)}^{2}+{\left(\frac{c_{\mathrm{EL}}^{\left(p\right)}}{K_{5}}\right)}^{2}+{\left(\frac{c_{\mathrm{CL}}^{\left(p\right)}}{K_{5b}}\right)}^{2}}‐k_{2}c_{P97}^{\left(m\right)},$$

$$\frac{dc_{P97}^{\left(p\right)}}{\mathrm{dt}}=p_{2}c_{P97}^{\left(m\right)}‐\left(d_{2D}D\left(t\right)+d_{2L}\;L\left(t\right)\right)c_{P97}^{\left(p\right)},$$

$$\frac{dc_{P51}^{\left(m\right)}}{\mathrm{dt}}=v_{3}\frac{1}{1+{\left(\frac{c_{\mathrm{CL}}^{\left(p\right)}}{K_{6}}\right)}^{2}+{\left(\frac{c_{P51}^{\left(p\right)}}{K_{7}}\right)}^{2}}‐k_{3}c_{P51}^{\left(m\right)},$$

$$\frac{dc_{P51}^{\left(p\right)}}{\mathrm{dt}}=p_{3}c_{P51}^{\left(m\right)}‐\left(d_{3D}D\left(t\right)+d_{3L}\;L\left(t\right)\right)c_{P51}^{\left(p\right)},$$

$$\frac{dc_{\mathrm{EL}}^{\left(m\right)}}{\mathrm{dt}}=L_{\mathrm{sens}}\;L\left(t\right)v_{4}\frac{1}{1+{\left(\frac{c_{\mathrm{CL}}^{\left(p\right)}}{K_{8}}\right)}^{2}+{\left(\frac{c_{P51}^{\left(p\right)}}{K_{9}}\right)}^{2}+{\left(\frac{c_{\mathrm{EL}}^{\left(p\right)}}{K_{10}}\right)}^{2}}‐k_{4}c_{\mathrm{EL}}^{\left(m\right)},$$

$$\frac{dc_{\mathrm{EL}}^{\left(p\right)}}{\mathrm{dt}}=p_{4}c_{\mathrm{EL}}^{\left(m\right)}‐\left(d_{4D}D\left(t\right)+d_{4L}L_{\mathrm{sens}}\;L\left(t\right)\right)c_{\mathrm{EL}}^{\left(p\right)},$$

$$\frac{\mathrm{dP}}{\mathrm{dt}}=0.3\left(1‐P\right)D\left(t\right)‐L_{\mathrm{sens}}\;L\left(t\right)P.$$

$c_{\alpha }^{\left(m\right)}$ and $c_{\alpha }^{\left(p\right)}$ represent the concentrations of the α‐th mRNA and protein (or protein complex) respectively, for *α* = CL, P97, P51, and EL. *P* represents the activity of the dark accumulated protein P. The parameters *v_j_*, *k_j_*, *p_j_*, and *d_j_* represent the reaction rates for transcription, mRNA degradation, translation, and protein degradation respectively, for the various reactions *j* = 1… (Appendix Table S1). *K_j_* represent a scaling factor for the protein concentrations. *D* represents a dark signal, which is equal to 1 when *L* = 0 and equal to 0 when *L* > 0. This explicit representation of a dark signal is commonly used in circadian clock models to capture the different reaction rates observed under dark conditions in experiments (Pokhilko *et al*, 2012; Fogelmark & Troein, 2014; De Caluwé *et al*, 2016).

### Parameter optimization

The original De Caluwé model contains 34 parameters, the values of which have been obtained previously through automated optimization (De Caluwé *et al*, 2016). Firstly, we adjusted the value of *d_3L_* to be less than *d_3D_*. We did this to account for the fact that several studies have demonstrated that the degradation of both *PRR5* and *TOC1* are increased in the dark (Más *et al*, 2003; Kiba *et al*, 2007; Kim *et al*, 2007; Fujiwara *et al*, 2008). We then used automated optimization to find the values related to the modified interaction between CL and P97 ($K_{0},\;K_{4},{\;K}_{5},$ and $K_{5b}$). We optimized by minimizing a cost function, combining Sobol search and simulated annealing to optimize within the range $K_{i}\in \left[0.1,10\right]$ for i=0,4,5,5b.

We computed the cost function as follows. First, we simulated the model under 12‐h light–12‐h dark conditions for a total of 60 days and then released to LL for 60 days, followed by constant darkness (DD) for 60 days. In each light condition, we discarded the first 55 days as a transient dynamic. To ensure detectable rhythmicity under LL and DD conditions, we required that all variables must have a minimum value of 0.1, as well as a minimum difference of 10% between their minimum and maximum values. We penalized any solution that did not meet these criteria with an arbitrarily large score. Then, we calculated the free‐running period from CL gene expression using the chi‐square periodogram (Sokolove & Bushell, 1978) at a significance level of 1%. We gave a score of 0 to a solution having a free‐running period between 24 and 25 h under LL and between 25 and 28 h under DD. For solutions with free‐running periods outside these ranges, we allocated the scores of 2 (*τ*_LL_ − 24.5)^2^/(0.1⋅24.5)^2^ and 2 (*τ*_DD_ − 26.5)^2^/(0.1⋅26.5)^2^, where *τ_LL_* and *τ_DD_* represent free‐running periods under LL and DD respectively. Under 12‐h light–12‐h dark cycles, we penalized solutions that did not entrain to the LD cycles with an arbitrarily large score. We assigned entrained solutions a score of 0 for each gene that attained peak expression within ± 1 h of the expected *ZT*, which were as follows: CL = 1.5, P97 = 6, P51 = 12, and EL = 9. We scored expression peaks lying outside these intervals as (*ZT_CL_* − 1.5)^2^/(0.1⋅24)^2^, (*ZT_P_*_97_ − 6)^2^/(0.1⋅24)^2^, (*ZT_P_*_51_ − 12)^2^/(0.1⋅24)^2^, (*ZT_EL_* − 9)^2^/(0.1⋅24)^2^, where *ZT* represents the peak time measured from the corresponding genes expression. Finally, we penalized solutions that showed a large phase shift, *Δ*, when transferred from LD cycles to LL. The phase shift was scored as *Δ* ^2^/(0.1⋅24)^2^. The optimized parameter values together with the original values determined by De Caluwé *et al* (2016) are listed in Appendix Table S1. The free‐running period of the revised model was 25.5 h under LL (*L* = 1; *D* = 0) and 27.4 h under constant darkness (*L* = 0; *D* = 1). Under LD cycles, the peak times (*ZT*) of the gene expressions were CL = −0.3 h, P97 = 6.0 h, P51 = 11.8 h, and EL = 9.7 h.

### Parameter sensitivity analysis

To test the robustness of the single cell model we carried out a parameter sensitivity analysis. We distributed all 34 parameters uniformly in the ± 5% range of their optimized value. For 50,000 sets of randomized parameter values, the free running period under LL (25.5 ± 0.4 h), free running period under DD (27.4 ± 0.5 h), amplitude under LL (0.9 ± 0.1), and amplitude under DD (1.4 ± 0.2) of CL expression were calculated. The values were distributed in a narrow range centered at their target values, indicating that they are not sensitive to the selected values of the 34 parameters (Appendix Fig S1). Our sensitivity analysis could in future be extended (e.g., by using Approximate Bayesian Computation) to calculate confidence intervals for the parameter estimates.

### Characterizing clock periods from experiments

We analyzed experimental luciferase and confocal data to characterize the periods within a seedling. We previously completed an organ‐level analysis of period and phase for *PRR9*::*LUC*, *ELF4*::*LUC*, and *TOC1*::*LUC* expression (Greenwood *et al*, 2019). In this analysis, 315 µm diameter regions of interest (ROI) were defined to represent the cotyledon, hypocotyl, root, and root tip regions of the seedling (Greenwood *et al*, 2022). These ROI were used to generate time series, from which period estimates were made across a number of individual seedlings. Here, we pooled estimates made from individual seedlings carrying *PRR9*::*LUC*, *ELF4*::*LUC*, or *TOC1*::*LUC* reporter genes, and calculated the mean for each region (Fig 2A). Only periods from time series classified as rhythmic (as defined previously (Greenwood *et al*, 2019)) were used in the calculation.

To characterize the variability of the periods, we measured the between‐cell variation of clock periods in each region of the plant (Appendix Fig S2). To do this we analyzed a dataset of single cell time‐lapse microscopy, *CCA1*::CCA1‐YFP fusion protein reporter data (Gould *et al*, 2018). We defined cells as being within the root tip region if they are less than 315 µm from the most distal cell of the root tip. Where there are multiple imaging sections in the same organ, cells are pooled to give one region for the cotyledon, hypocotyl, and root. Variability was estimated using the coefficient of variation (CV; SD/mean) of all cells within these regions. Only periods from time series classified as rhythmic (as defined previously (Gould *et al*, 2018)) were used in the calculation.

### Spatial molecular model

We implemented our optimized molecular model on a simplified template of a seedling. The template consisted of approximately 800 cells, classified into cotyledon, hypocotyl, root, and root tip regions (Fig 1B). Each cell contained an implementation of the revised compact model. To simulate growth of the seedlings, we added a row of cells to the root tip every 24 h. During this growth, we kept the root tip region of the template fixed in size. To do this, after the addition of new cells, the previously uppermost root tip cells became root cells instead.

We set the light sensitivity of the cell, *L_sens_*, to vary depending on the position within the template. In doing so, we found that modest differences in *L_sens_* were sufficient to generate period differences between regions. To approximate the period differences that we observed between regions in experiments (Fig 2A) we set *L_sens_* = 1.6 for cotyledon cells, *L_sens_* = 1.0 for hypocotyl cells, *L_sens_* = 0.65 for root cells, and *L_sens_* = 0.95 for root tip cells. As the template grows, the uppermost root tip cells become root cells, so the light sensitivity of these cells decreases so that they have a sensitivity characteristic of root cells, *L_sens_* = 0.65. This caused the clock period of these cells to slow.

As the coupling agents have yet to be clearly identified by experimental studies, we initially assumed individual cells to be coupled through CL. Our model for coupled plant cells is described as follows:

$$\tau_{i}\frac{dc_{\mathrm{CL},i}^{\left(m\right)}}{\mathrm{dt}}=\left(v_{1\;}+v_{1L}L_{\mathrm{sens},i}\;L\left(t\right)P\right)\frac{1}{1+{\left(\frac{c_{\mathrm{CL}}^{\left(p\right)}}{K_{0}}\right)}^{2}+{\left(\frac{c_{P97}^{\left(p\right)}}{K_{1}}\right)}^{2}+{\left(\frac{c_{P51}^{\left(p\right)}}{K_{2}}\right)}^{2}}\;‐\left(k_{1L}L_{\mathrm{sens},i}\;L\left(t\right)+k_{1D}D\left(t\right)\right)c_{\mathrm{CL}}^{\left(m\right)}+\;J_{\mathrm{local}}\left({\bar{c}}_{CL,i}^{\left(m\right)}‐c_{CL,i}^{\left(m\right)}\right),$$

$$\tau_{i}\;\frac{dc_{CL,i}^{\left(p\right)}}{\mathrm{dt}}=\left(p_{1}+p_{1L}L_{sens,i}\;L\left(t\right)\right)c_{CL,i}^{\left(m\right)}‐d_{1}c_{CL,i}^{\left(p\right)},$$

$$\tau_{i}\;\frac{dc_{P97,i}^{\left(m\right)}}{\mathrm{dt}}=\left(v_{2L}L_{sens,i}\;L\left(t\right)P+v_{2A}\right)\frac{1}{1+{\left(\frac{c_{P51}^{\left(p\right)}}{K_{4}}\right)}^{2}+{\left(\frac{c_{\mathrm{EL}}^{\left(p\right)}}{K_{5}}\right)}^{2}+{\left(\frac{c_{\mathrm{CL}}^{\left(p\right)}}{K_{5b}}\right)}^{2}}\;‐k_{2}c_{P97,i}^{\left(m\right)},$$

$$\tau_{i}\;\frac{dc_{P97,i}^{\left(p\right)}}{\mathrm{dt}}=p_{2}c_{P97,i}^{\left(m\right)}‐\left(d_{2D}D\left(t\right)+d_{2L}\;L\left(t\right)\right)c_{P97,i}^{\left(p\right)},$$

$$\tau_{i}\;\frac{dc_{P51,i}^{\left(m\right)}}{\mathrm{dt}}=v_{3}\frac{1}{1+{\left(\frac{c_{\mathrm{CL}}^{\left(p\right)}}{K_{6}}\right)}^{2}+{\left(\frac{c_{P51}^{\left(p\right)}}{K_{7}}\right)}^{2}}‐k_{3}c_{P51,i}^{\left(m\right)},$$

$$\tau_{i}\;\frac{dc_{P51,i}^{\left(p\right)}}{\mathrm{dt}}{=p}_{3}c_{P51,i}^{\left(m\right)}‐\left(d_{3D}D\left(t\right)+d_{3L}\;L\left(t\right)\right)c_{P51,i}^{\left(p\right)},$$

$$\tau_{i}\;\frac{dc_{EL,i}^{\left(m\right)}}{\mathrm{dt}}=L_{sens,i}\;L\left(t\right)v_{4}\frac{1}{1+{\left(\frac{c_{\mathrm{CL}}^{\left(p\right)}}{K_{8}}\right)}^{2}+{\left(\frac{c_{P51}^{\left(p\right)}}{K_{9}}\right)}^{2}+{\left(\frac{c_{\mathrm{EL}}^{\left(p\right)}}{K_{10}}\right)}^{2}}‐k_{4}c_{EL,i}^{\left(m\right)},$$

$$\tau_{i}\;\frac{dc_{EL,i}^{\left(p\right)}}{\mathrm{dt}}=p_{4}c_{EL,i}^{\left(m\right)}‐\left(d_{4D}D\left(t\right)+d_{4L}L_{sens,i}\;L\left(t\right)\right)c_{EL,i}^{\left(p\right)}\;+J_{\mathrm{long}}\left({\bar{c}}_{EL,long}^{\left(p\right)}‐c_{EL,i}^{\left(p\right)}\right),$$

$$\tau_{i}\;\frac{\mathrm{dP}}{\mathrm{dt}}=0.3\left(1‐P\right)D\left(t\right)‐L_{sens,i}\;L\left(t\right)P.$$

As in the revised single‐cell model, $c_{\alpha }^{\left(m\right)}$ and $c_{\alpha }^{\left(p\right)}$ represent the concentration of the *α*‐th mRNA and protein (or protein complex) respectively for *α* = CL, P97, P51, and EL, in the *i*‐th cell (i=1,2,…,N). *P* again represents activity of the dark accumulated protein P. To generate cell‐to‐cell variability in periods, we multiply the derivative with respect to the time of each molecular species by the time scaling parameter *τ*. *τ* is a real number randomly selected from the normal distribution N(1, 0.059), N(1, 0.028), N(1, 0.073), N(1, 0.089) for cells from the cotyledon, hypocotyl, root, or root tip respectively, as informed by the analysis of single‐cell experimental data (see “Characterizing clock periods from experiments”).

In the first equation, the CL gene is locally coupled to its neighboring cells, where *J_local_* is the coupling strength and ${\bar{c}}_{CL,i}^{\left(m\right)}=\frac{1}{4}\sum_{j\in N\left(i\right)}c_{CL,j}^{\left(m\right)}$ represents averaged expression level over 4 neighboring cells (left, right, above, and below). In the case of local coupling between 8 neighboring cells (left, right, above, below, left above, left below, right above, and right below) or global coupling, the averaged expression becomes ${\bar{c}}_{CL,i}^{\left(m\right)}=\frac{1}{8}\sum_{j\in N\left(i\right)}c_{CL,j}^{\left(m\right)}$ or ${\bar{c}}_{CL,i}^{\left(m\right)}=\frac{1}{N}\sum_{j=1}^{N}c_{CL,j}^{\left(m\right)}$ respectively. In Fig EV2 we varied the identity of the local coupling component. Here, the averaged expression becomes ${\bar{c}}_{\alpha ,i}^{\left(m\right)}=\frac{1}{4}\sum_{j\in N\left(i\right)}c_{\alpha ,j}^{\left(m\right)}$ or ${\bar{c}}_{\alpha ,i}^{\left(p\right)}=\frac{1}{4}\sum_{j\in N\left(i\right)}c_{\alpha ,j}^{\left(p\right)}$ for coupling through mRNA or protein respectively, for *α* = CL, P97, P51, EL. The coupling term is appended to the equation of the corresponding molecular species. We set the local coupling strength as *J_local_* = 0, 0.01, 0.1, 1, 2, or 4.

In Fig 4, we simulated long‐distance coupling between the hypocotyl and root tip cells through the movement of ELF4. To implement this, a long‐distance coupling term is appended to the equations describing EL protein in root tip cells, where *J_long_* is the long‐distance coupling strength and ${\bar{c}}_{EL,long}^{\left(p\right)}=\frac{1}{N_{R}}\sum_{j\in R}c_{EL,j}^{\left(p\right)}$ represents the averaged expression level over cells in the hypocotyl, *R*. We set the long‐distance coupling strength as *J_long_* = 0, 0.01, 0.1, 1, 2, or 4.

### Analysis of peaks and periods of expression

To assess our model, we quantified the times of the peaks and peak‐to‐peak times of simulated gene expression. We firstly did this from ROI averages to allow for comparison to experimental data (e.g., Fig 2) (Greenwood *et al*, 2019). We defined 5‐by‐3 (width‐by‐height) pixel ROI on the seedling template, approximating the positions used in the experimental analysis (Appendix Fig S7A). We took the mean of these ROI at each time point to give the simulated time series. We identified the peaks of the time series within 24–144 h after transfer to LL using the “findpeaks” MATLAB (MathWorks, UK) function. We imposed that peaks must be more than 19 h apart and greater than the mean of the time series. Next, we measured the period as the mean of the times between consecutive peaks. In Figs 2 and 3, we plotted the analysis of the simulated data with previously collected experimental data (Greenwood *et al*, 2019) for comparison. For visual clarity, only peaks in which all organs completed the full cycle within the time window were plotted. Additionally, only peaks from the experimental time series classified as rhythmic (as defined previously (Greenwood *et al*, 2019)) were plotted.

### Space‐time intensity plots

To visualize the spatial clock dynamics, we created space‐time intensity plots of experimental and simulated gene expression (e.g., Fig 3A and C). We made plots of *PRR9*::*LUC*, *ELF4*::*LUC*, and *TOC1*::*LUC* expression as described previously (Greenwood *et al*, 2019). In brief, we average expression across sections that are perpendicular to the primary axis of the seedling. For simulations, we take the mean expression from 1‐by‐5 cell cross‐sections through the primary axis of the template (Appendix Fig S7B, yellow lines). We additionally plotted the times of the final peaks of expression of the space‐time plots (e.g., Fig 3B and D). We detected peaks as before (see “Analysis of peaks and periods of expression”), but restricted the detection to within a 24 h window of the expected time of the final peak of expression. If more than one peak was detected we plotted the peak with the greatest height. However, under some conditions, we consistently observed two peaks of *TOC1* expression within 24 h. We therefore plotted the earliest peak of *TOC1* expression.

### Simulations under LD cycles

We simulated light‐dark cycles with a day length of 8, 12, or 16 h. We termed these cycles without noise “idealized LD” (Fig 5A, left). To simulate fluctuations in the light cycle we utilized an algorithm developed previously (Pittayakanchit *et al*, 2018), which approximates meteorological data (Gu *et al*, 2001). Fluctuations lasted an interval of time drawn randomly from an exponential distribution of mean 2.4 h. The intensity of light during each fluctuation was drawn randomly from a second function of uniform distribution in the range 0.5 to 1.5. We termed this condition “noisy LD” (Fig 5A, right). LD cycles were input to the molecular model through the parameter *L*.

Individual plant cells may not experience a unique noisy LD cycle but instead have some correlation in the light cycle between one another. To examine the dependence of our results on the correlation, we extended our algorithm to consider two types of fluctuations, global and individual. The global fluctuation, *ξ_global_*, is applied commonly to all cells, while the individual fluctuation, *ξ_individual_,* is applied only to individual cells. The global and individual fluctuations are drawn randomly from the same distributions as before, but are statistically independent from each other. By combining the two fluctuations, each cell receives a light input as $L\left(t\right)={1‐\{\;\lambda \;\xi }_{\mathrm{global}}\left(t\right)+{(1‐\lambda )\;\xi }_{\mathrm{individual}}\left(t\right)\;\}$ with a mixture ratio of 0≤λ≤1. If λ=1, the same fluctuation is applied to all the cells. If on the other hand λ=0, fluctuations applied to individual cells are all independent from each other. We simulate noisy LD cycles with *λ* = 0, 0.1, 0.25, 0.5, and 1.

### Timing error analysis

We introduced the cell timing error as a relative measure of clock precision under noisy LD cycles. For all cells of a simulation (*h* = 1, 2 ,..., *N*), we computed the times of the final peaks of expression *T*. We computed the peaks under the noisy LD condition *T_noisy_,* and the idealized LD condition, *T_idealized_*, with a matching strength of local coupling. The latter condition acted as a benchmark for the cell’s ideal time of expression, in order to estimate the error caused by the noise. We then calculate the timing error *E* at each coupling strength as

$$E=\frac{1}{N}\sum_{h=1}^{N}\left|T_{\mathrm{idealized}}^{h}\;‐T_{\mathrm{noisy}}^{h}\right|.$$

### Statistical methods

In the experimental analysis, we excluded seedlings from the analysis if they were not rhythmic, as described in the previous experimental studies (Mockler *et al*, 2007; Gould *et al*, 2018; Greenwood *et al*, 2019). We plotted the mean and SD when the results of the analysis were normally distributed, and otherwise plotted the median, 25‐th, and 75‐th percentile. We chose representative seedlings for the space‐time intensity plots. For simulations, we performed 9 simulations under each condition. We performed each simulation with a different distribution of the time scaling parameter, τ, (see “Spatial molecular model”). We matched these distributions between different conditions to allow for comparison. Summary statistics and representative space‐time intensity plots were presented, as for experiments. We did not use statistical tests to select the sample size or perform blinding during the analysis.

## Acknowledgements

MG and JL were supported by the Gatsby Charitable Foundation (grant number GAT3395/GLC) and IT by the Japan Society for the Promotion of Science (grant numbers 17H06313, 18H02477, and 20K11875). We thank Dr. Katie Abley (University of Cambridge) for critical reading of the manuscript.

## Author contributions

**Mark Greenwood:** Conceptualization; Resources; Data curation; Software; Formal analysis; Validation; Investigation; Visualization; Methodology; Writing—original draft; Project administration; Writing—review and editing. **Isao T Tokuda:** Conceptualization; Resources; Software; Formal analysis; Supervision; Funding acquisition; Validation; Investigation; Visualization; Methodology; Project administration; Writing—review and editing. **James C W Locke:** Conceptualization; Supervision; Funding acquisition; Writing—original draft; Project administration; Writing—review and editing.

In addition to the CRediT author contributions listed above, the contributions in detail are:

MG and ITT performed the simulations. MG analyzed the experimental data. MG, IT, and JCWL designed the study and wrote the manuscript.

## Disclosure and competing interests statement

The authors declare that they have no conflict of interest.

## Supporting information



Appendix

Expanded View Figures PDF

Movie EV1

Movie EV2

## Data Availability

Source data for main and extended view figures, as well as the model code produced in this study is available from our project GitLab page (https://gitlab.com/slcu/teamJL/greenwood_etal_2022).
