# Supplementary material for: A spatial model of the plant circadian clock reveals design principles for coordinated timing
Source: Mol Syst Biol. 2022 Mar 21;18(3):e10140. doi: 10.15252/msb.202010140 (PMC8935279; doi:10.15252/msb.202010140)
Supplement: Supplementary file 1 — Appendix [file MSB-18-e10140-s005.pdf]

Appendix

Table of contents

Appendix Figure S1. Parameter sensitivity of the modified network model.

Appendix Figure S2. Between-cell variability of CCA1-YFP rhythms.

Appendix Figure S3. Simulated expression of clock genes in different regions under LD cycles.

Appendix Figure S4. Simulated expression of clock genes under LL qualitatively match luciferase reporter experiments.

Appendix Figure S5. Simulations with higher light sensitivity show shorter periods.

Appendix Figure S6. Local sharing of clock molecules can reproduce the experimentally observed spatial waves of multiple clock genes.

Appendix Figure S7. Spatial analysis of rhythms on the plant template.

Appendix Figure S8. Peaks of simulated expression under LL with increasing strengths of coupling.

Appendix Figure S9. Peaks of simulated expression under LL without cell-to-cell variability in gene expression.

Appendix Figure S10. Peaks of simulated expression under LD cycles.

Appendix Figure S11. Peaks of simulated expression under different day lengths.

Appendix Figure S12. Simulated expression under noisy LD cycles.

Appendix Figure S13. Cell timing error with correlated noise in the LD cycles.

Appendix Table S1. Parameter values for the spatial clock model.

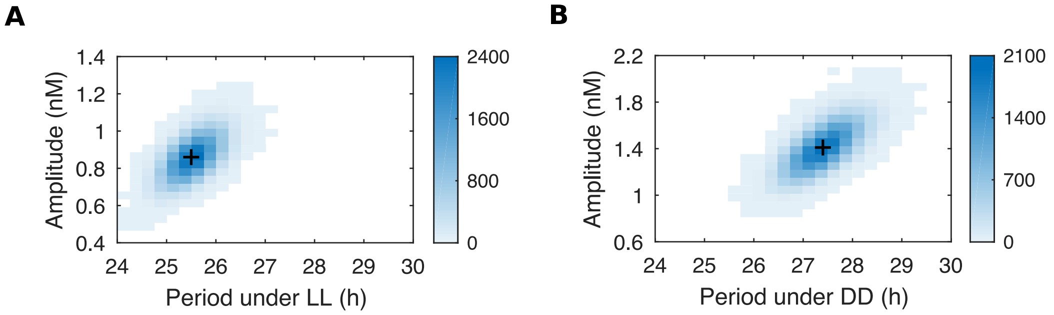

Appendix Figure S1. Parameter sensitivity of the modified network model

A, B. All 34 parameters were uniformly distributed in the ± 5 % range of the optimal values used in the model. For 50,000 sets of parameters, the free running periods and amplitudes under LL (A) or constant dark (B) were measured. The color represents the bin count and the black cross the value of the optimal parameter set used for the model. See Materials and Methods for details.

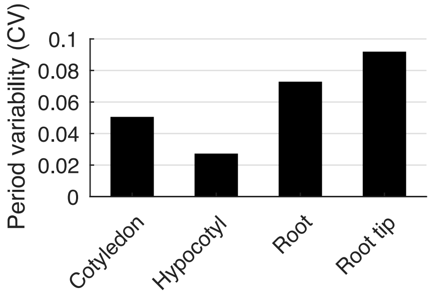

Appendix Figure S2. Between cell variability of CCA1-YFP rhythms

Between-cell variability of periods within different regions of a single *Arabidopsis* seedling.

Data information: The periods were estimated from single cell time-lapse movies of CCA1-YFP expression carried out previously [8]. For the cotyledon, *n* = 103; hypocotyl, *n* = 115; root, *n* = 144; root tip, *n* = 280. *n* represents the number of rhythmic cells from 1 independent experiment.

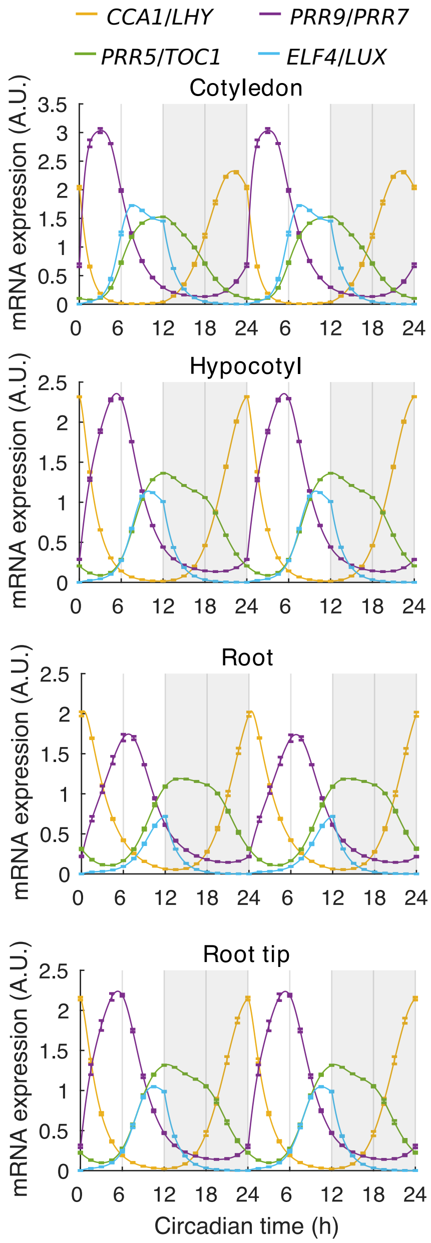

Appendix Figure S3. Simulated expression of clock genes in different regions under LD cycles

Expression of simulated *CCA1*/*LHY*, *PRR9*/*PRR7*, *PRR5*/*TOC1*, and *ELF4*/*LUX* from regions of the seedling template 96–144 h after the beginning of LD cycles. The light sensitivity, L_{sens}, was varied depending on whether the cell was within the cotyledon (L_{sens} = 1.6), hypocotyl (L_{sens} = 1.0), root (L_{sens} = 0.65), or root tip (L_{sens} = 0.95) region, and local cell-to-cell coupling was

present between cells (J_{local} = 2). Data points represent the mean and error bars ± the standard error, n = 9 simulations. Error bars are shown at 1.5 h intervals.

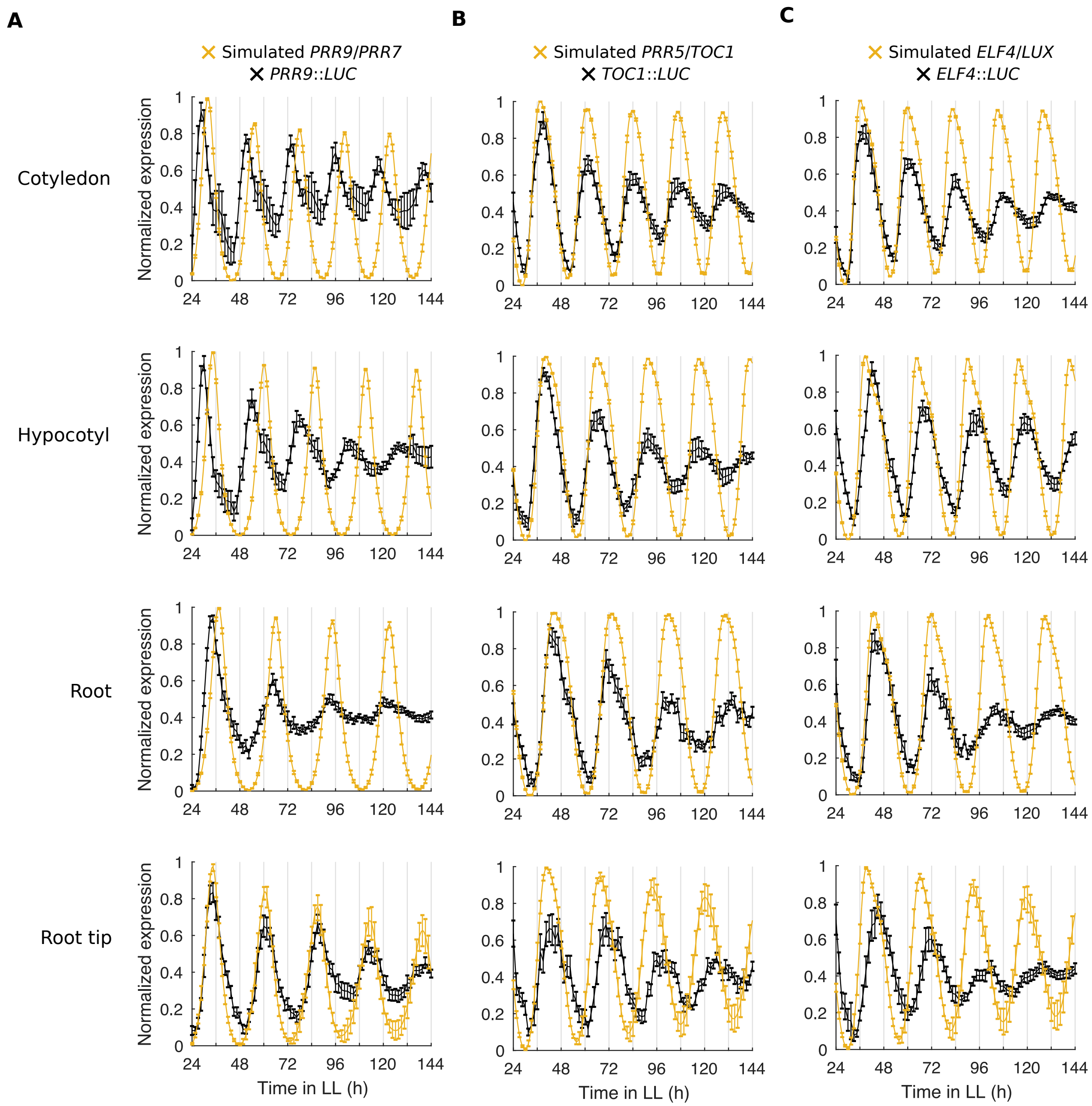

Appendix Figure S4. Simulated expression of clock genes under LL qualitatively match luciferase reporter experiments

A–C. Expression of simulated *PRR9*/*PRR7* and *PRR9*::*LUC* (A), simulated *PRR5*/*TOC1* and *TOC1*::*LUC (B)*, or simulated *ELF4*/*LUX* and *ELF4*::*LUC* (C) in different organs under LL. In simulations the light sensitivity was varied between regions and local cell-to-cell coupling was

present (J_{local} = 2). Data points represent the mean and error bars ± the standard error of organs scored as rhythmic. *n* = 9 simulations. Error bars are shown at 1.5 h intervals.

Data information: Experimental data is from *Arabidopsis* time-lapse movies carried out previously [14]. For *PRR9*::*LUC* data *N* = 4; *TOC1*::*LUC* data *N* = 3; *ELF4*::*LUC* data *N* = 3. For all, *n* = 7−18. *N* represents the number of independent experiments and *n* the total number of organs tracked.

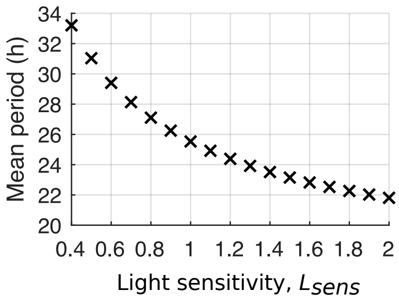

Appendix Figure S5. Simulations with higher light sensitivity show shorter periods

Mean period estimates of simulated *PRR9*/*PRR7* expression with increasing sensitivity to light, L_{sens}. A single cell implementation of the model was simulated at each L_{sens}, without cell-to-cell coupling or variation in gene expression. L_{sens} values below approximately 0.8 in the original De Caluwé and below 0.4 in our modified implementation resulted in arrhythmicity.

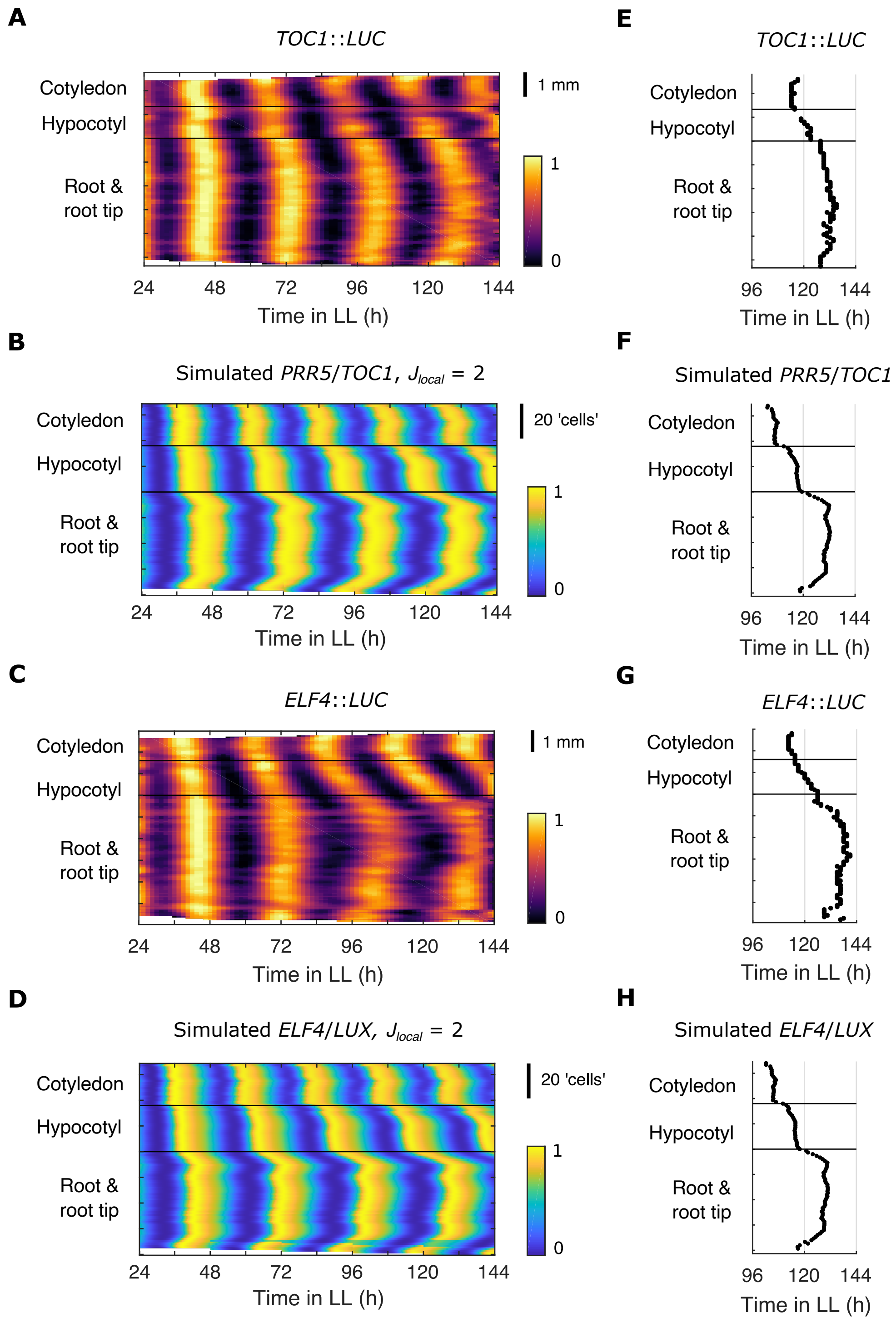

Appendix Figure S6. Local sharing of clock molecules can reproduce the experimentally observed spatial waves of multiple clock genes

A–D. Representative intensity plot of *TOC1*::*LUC* (A), simulated *PRR5*/*TOC1* (B), *ELF4*::*LUC* (C) and simulated *ELF4*/*LUX* (D) expression measured from longitudinal sections of a single

seedling under LL. Simulations assumed varied light sensitivities and local cell-to-cell coupling (J_{local} = 2).

E–H. Times of the final peaks of *TOC1*::*LUC* (E), simulated *PRR5*/*TOC1* (F)*, ELF4*::*LUC* (G) and simulated *ELF4*/*LUX* (H) intensity plots.

Data information: Experimental data is from *Arabidopsis* time-lapse movies carried out previously [14]. For *TOC1*::*LUC* data *N* = 3; *ELF4*::*LUC* data *N* = 3. For both, *n* = 10−18. *N* represents the number of independent experiments and *n* the total number of organs tracked. Data in F and H are replotted as 'J_{local} = 2' within Appendix Fig S8B and C respectively.

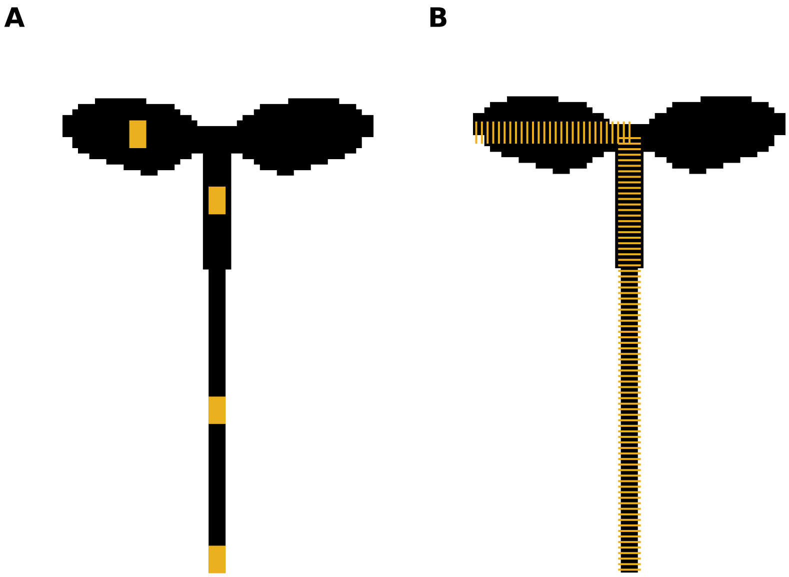

Appendix Figure S7. Spatial analysis of rhythms on the plant template

A. For comparison to experimental data simulated we extracted expression from 3-by-3-pixel regions of interest of the template (yellow squares).

B. To produce space-time plots of gene expression (e.g. Fig 3C), we take the mean expression from 1-by-5 cell sections (yellow lines) perpendicular to the central axis of the template.

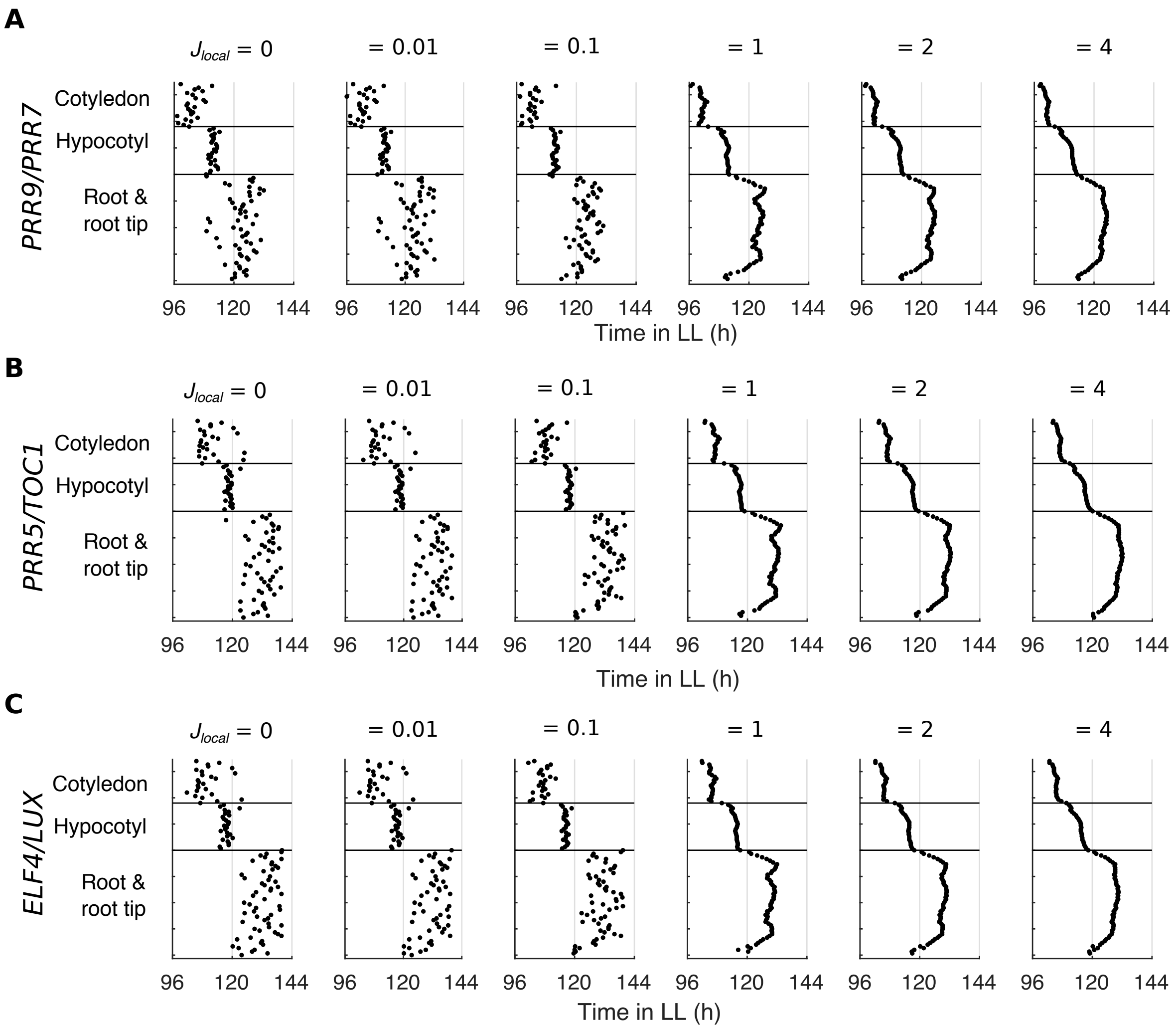

Appendix Figure S8. Peaks of simulated expression under LL with increasing strengths of coupling

A–C. The times of the final peaks of simulated *PRR9/PRR7* (A), *PRR5*/*TOC1* (B), and *ELF4*/*LUX* (C) intensity plots, each simulated under LL with increasing strengths of local cell-to-cell coupling.

Data information: A is replotted from Fig 3E. Data in B and C ('J_{local} = 2') are replotted from Appendix Fig 6F, and Appendix Fig 6H respectively.

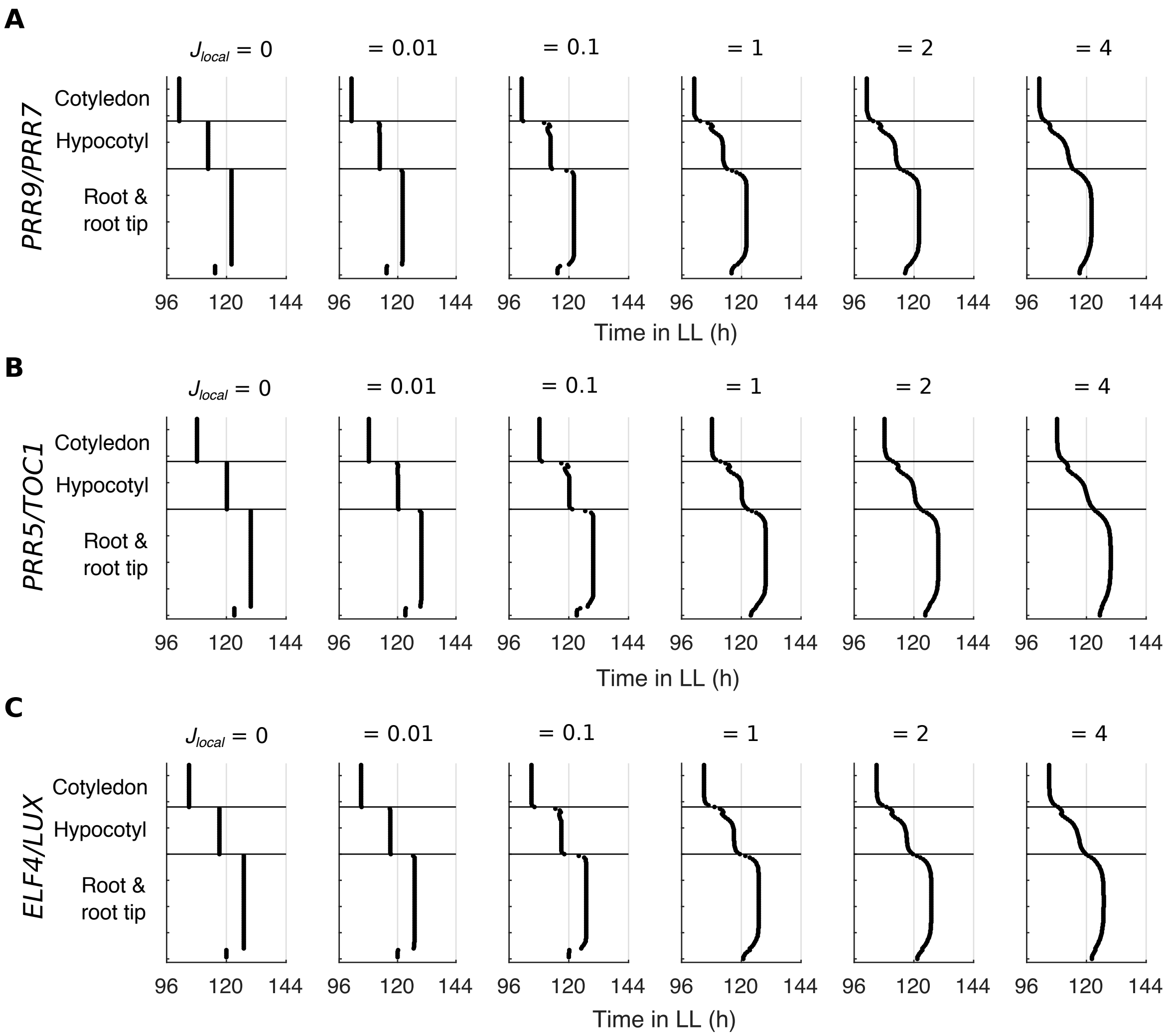

Appendix Figure S9. Peaks of simulated expression under LL without cell-to-cell variability in gene expression.

A–C. The times of the final peaks of simulated *PRR9*/*PRR7* (A), *PRR5*/*TOC1* (B), or *ELF4*/*LUX* (C) intensity plots, simulated under LL with increasing strengths of local cell-to-cell coupling, but without cell-to-cell variation in gene expression.

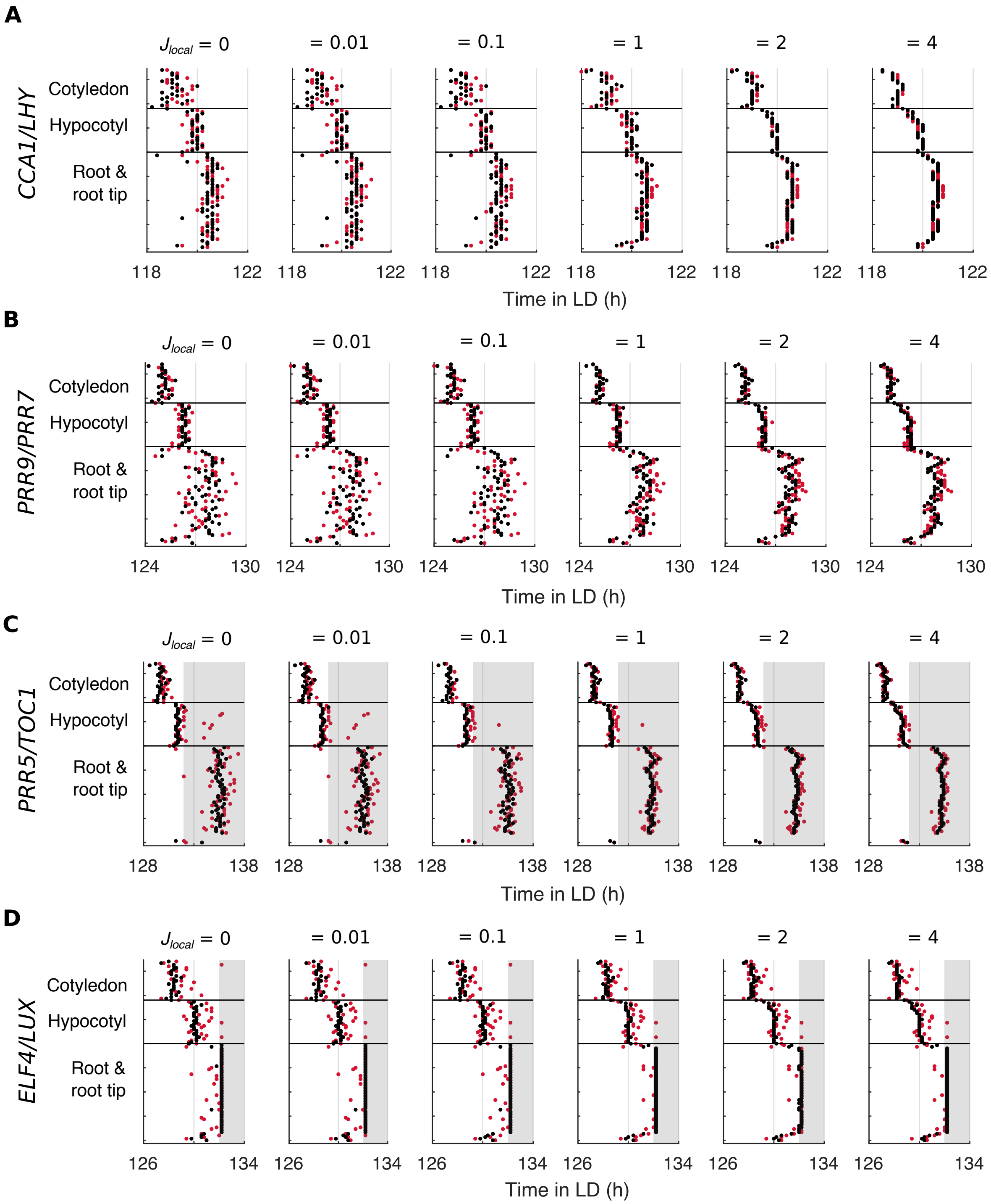

Appendix Figure S10. Peaks of simulated expression under LD cycles

A–D. The times of the final peaks of simulated *CCA1*/*LHY* (A), *PRR9*/*PRR7* (B), *PRR5*/*TOC1* (C), and *ELF4*/*LUX* (D) intensity plots, simulated under idealized (black dots) or noisy (red dots)

LD cycles. Simulations were performed with increasing strengths of local cell-to-cell coupling. The grey background indicates nighttime.

Data information: Data in A, C, and D (‘J_{local} = 2’) are replotted within Appendix Fig S11 A, C, and D respectively as ’12-h day’. B is replotted from Fig 5B.

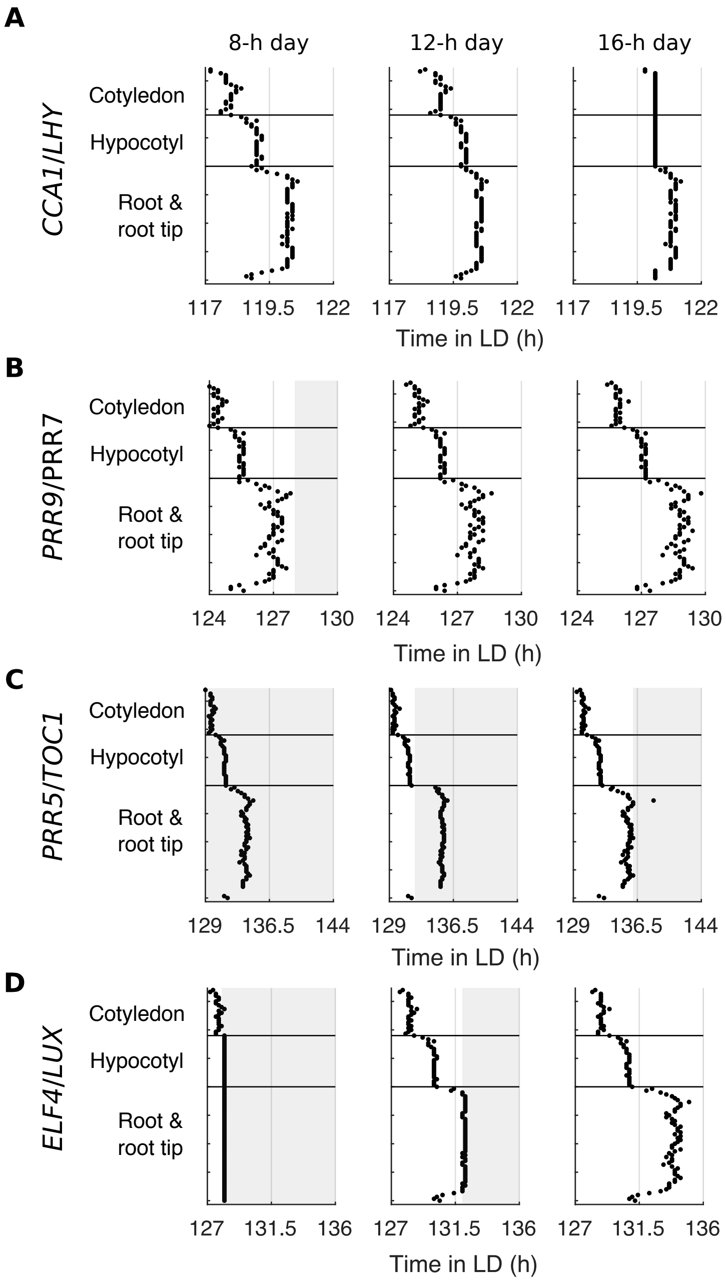

Appendix Figure S11. Peaks of simulated expression under different day lengths

A–D. The times of the final peaks of simulated *CCA1*/*LHY* (A), *PRR9*/*PRR7* (B), *PRR5*/*TOC1* (C), and *ELF4*/*LUX* (D) intensity plots, simulated under idealized LD cycles with 8 h, 12 h, or 16 h day lengths, and with local cell-to-cell coupling (J_{local} = 2).

Data information: Data in A, C, and D ('12-h day') are replotted from Appendix Fig S10 A, C, and D respectively ('J_{local} = 2'). Data in B ('12-h day') is replotted from Fig 5B ('J_{local} = 2').

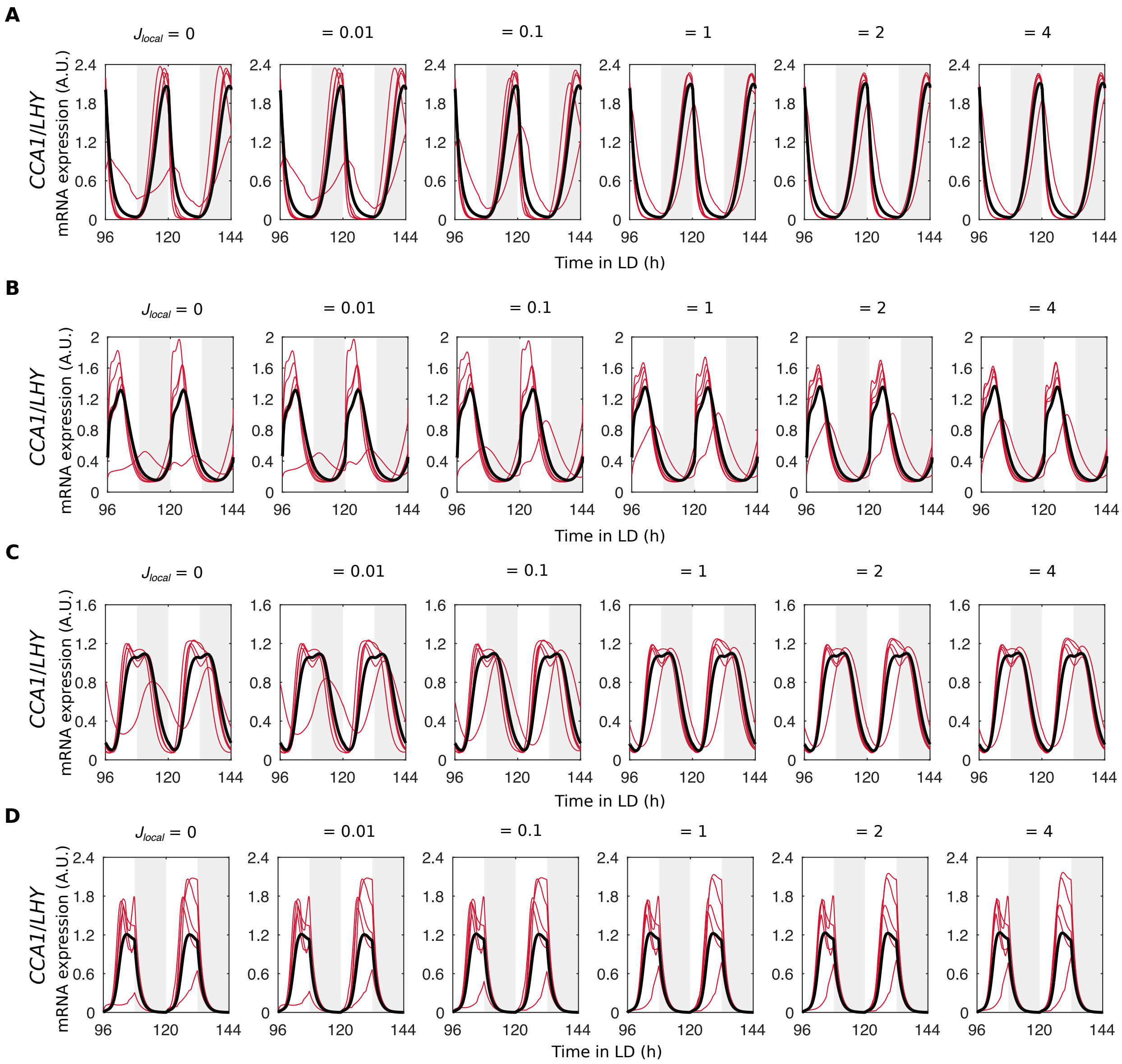

Appendix Figure S12. Simulated expression under noisy LD cycles

A–D. Expression of simulated *CCA1*/*LHY* (A), *PRR9*/*PRR7* (B), *PRR5*/*TOC1* (C), and *ELF4*/*LUX* (D) performed under noisy LD cycles and with increasing strengths of local cell-to-cell coupling. For each condition, 5 representative cells (red lines) and the mean of all cells in a single simulation (black line) were plotted.

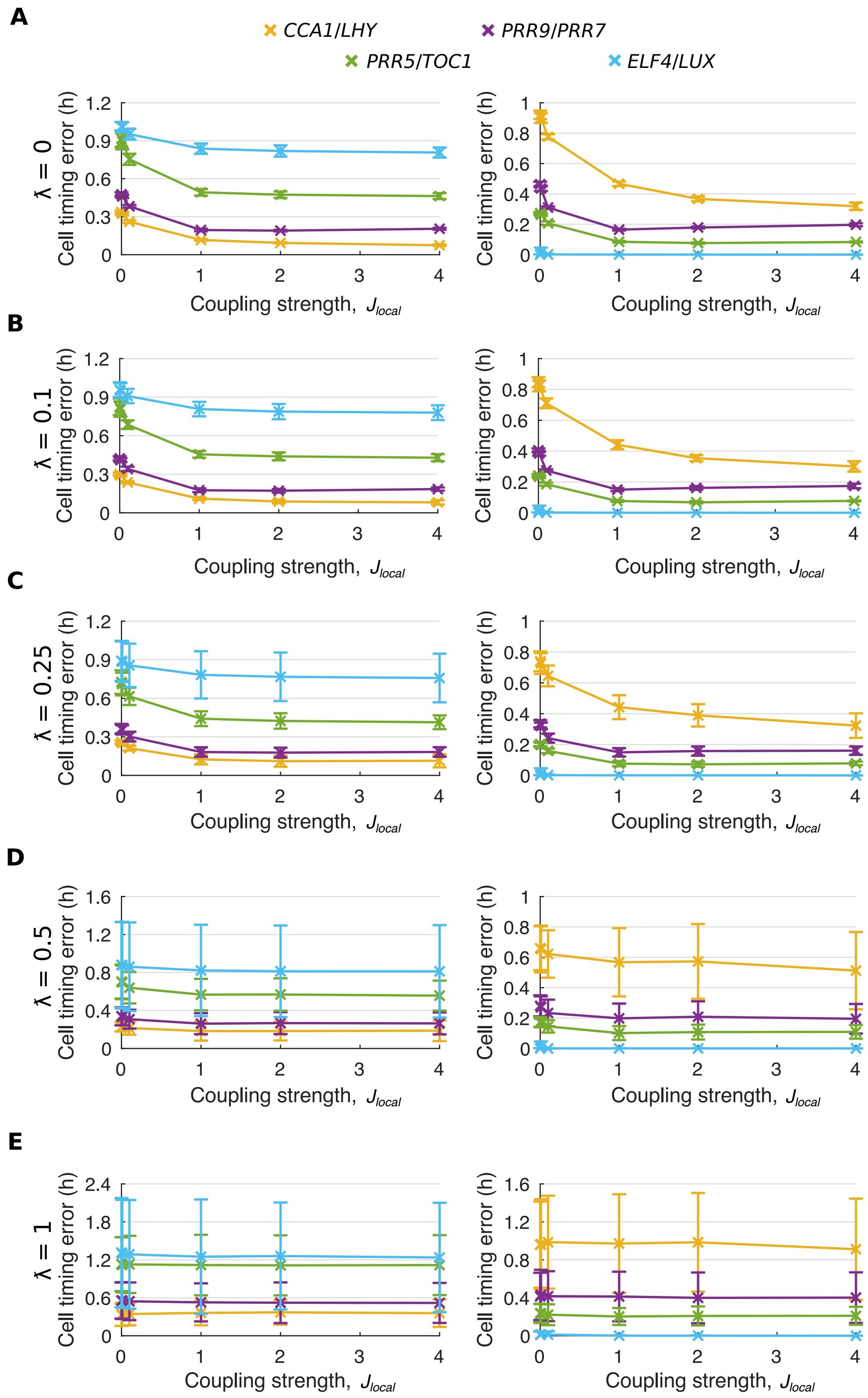

Appendix Figure S13. Cell timing error with correlated noise in the LD cycles

(A–E) The cell timing error calculated from the peaks (left) or troughs (right) of simulated gene expression, with increasing strengths of local cell-to-cell coupling. The correlation between the noisy LD cycles was varied according to ƛ, so that cells experienced independent cycles ƛ = 0 (A), correlated cycles ƛ = 0.10 (B), ƛ = 0.25 (C), ƛ = 0.50 (D), or identical cycles ƛ = 1 (E). Data points represent the mean ± standard deviation, n = 9 simulations.

Data information: A is replotted from Fig 5C and D.

Appendix Table S1. Parameter values for the spatial clock model.

Parameter	Description	Original value [32]	Re-optimized value
v_1	*CCA1*/*LHY* synthesis	4.58 nM h^{-1}	4.58 nM h^{-1}
v_{1L}	*CCA1*/*LHY* light-induced synthesis	3.0 nM h^{-1}	3.0 nM h^{-1}
v_{2A}	*PRR9*/*PRR7* synthesis	1.27 nM h^{-1}	1.27 nM h^{-1}
v_{2L}	*PRR9*/*PRR7* light-induced synthesis	5.0 nM h^{-1}	5.0 nM h^{-1}
v_3	*PRR5*/*TOC1* synthesis	1.0 nM h^{-1}	1.0 nM h^{-1}
v_4	*ELF4*/*LUX* synthesis	1.47 nM h^{-1}	1.47 nM h^{-1}
k_{1L}	*CCA1*/*LHY* mRNA degradation (light)	0.53 h^{-1}	0.53 h^{-1}
k_{1D}	*CCA1*/*LHY* mRNA degradation (dark)	0.21 h^{-1}	0.21 h^{-1}
k_2	*PRR9*/*PRR7* mRNA degradation	0.35 h^{-1}	0.35 h^{-1}
k_3	*PRR5*/*TOC1* mRNA degradation	0.56 h^{-1}	0.56 h^{-1}
k_4	*ELF4*/*LUX* mRNA degradation	0.57 h^{-1}	0.57 h^{-1}
p_1	CCA1/LHY translation	0.76 h^{-1}	0.76 h^{-1}
p_{1L}	CCA1/LHY light-induced translation	0.42 h^{-1}	0.42 h^{-1}
p_2	PRR9/PRR7 translation	1.01 h^{-1}	1.01 h^{-1}
p_3	PRR5/TOC1 translation	0.64 h^{-1}	0.64 h^{-1}
p_4	ELF4/LUX translation	1.01 h^{-1}	1.01 h^{-1}
d_1	CCA1/LHY degradation	0.68 h^{-1}	0.68 h^{-1}
d_{2D}	PRR9/PRR7 degradation (dark)	0.50 h^{-1}	0.50 h^{-1}
d_{2L}	PRR9/PRR7 degradation (light)	0.29 h^{-1}	0.29 h^{-1}
d_{3D}	PRR5/TOC1 degradation (dark)	0.48 h^{-1}	0.48 h^{-1}
d_{3L}	PRR5/TOC1 degradation (light)	0.78 h^{-1}	0.38 h^{-1}
d_{4D}	ELF4/LUX degradation (dark)	1.21 h^{-1}	1.21 h^{-1}

d_{4L}	ELF4/LUX degradation (light)	0.38 h^{-1}	0.38 h^{-1}
K_0	Inhibition of CCA1/LHY by CCA1/LHY	n/a	2.80 nM
K_1	Inhibition of CCA1/LHY by PRR9/PRR7	0.16 nM	0.16 nM
K_2	Inhibition of CCA1/LHY by PRR5/TOC1	1.18 nM	1.18 nM
K_4	Inhibition of PRR9/PRR7 by PRR5/TOC1	0.23 nM	0.28 nM
K_5	Inhibition of PRR9/PRR7 by ELF4/LUX	0.30 nM	0.57 nM
K_{5b}	Inhibition of PRR9/PRR7 by CCA1/LHY	n/a	1.73 nM
K_6	Inhibition of PRR5/TOC1 by CCA1/LHY	0.46 nM	0.46 nM
K_7	Inhibition of PRR5/TOC1 by PRR5/TOC1	2.0 nM	2.0 nM
K_8	Inhibition of ELF4/LUX by CCA1/LHY	0.36 nM	0.36 nM
K_9	Inhibition of ELF4/LUX by PRR5/TOC1	1.9 nM	1.9 nM
K_{10}	Inhibition of ELF4/LUX by ELF4/LUX	1.9 nM	1.9 nM
