# Supplementary material for: A spatial model of the plant circadian clock reveals design principles for coordinated timing
Source: Mol Syst Biol. 2022 Mar 21;18(3):e10140. doi: 10.15252/msb.202010140 (PMC8935279; doi:10.15252/msb.202010140)
Supplement: Supplementary file 2 — Expanded View Figures PDF [file MSB-18-e10140-s004.pdf]

Expanded View Figures

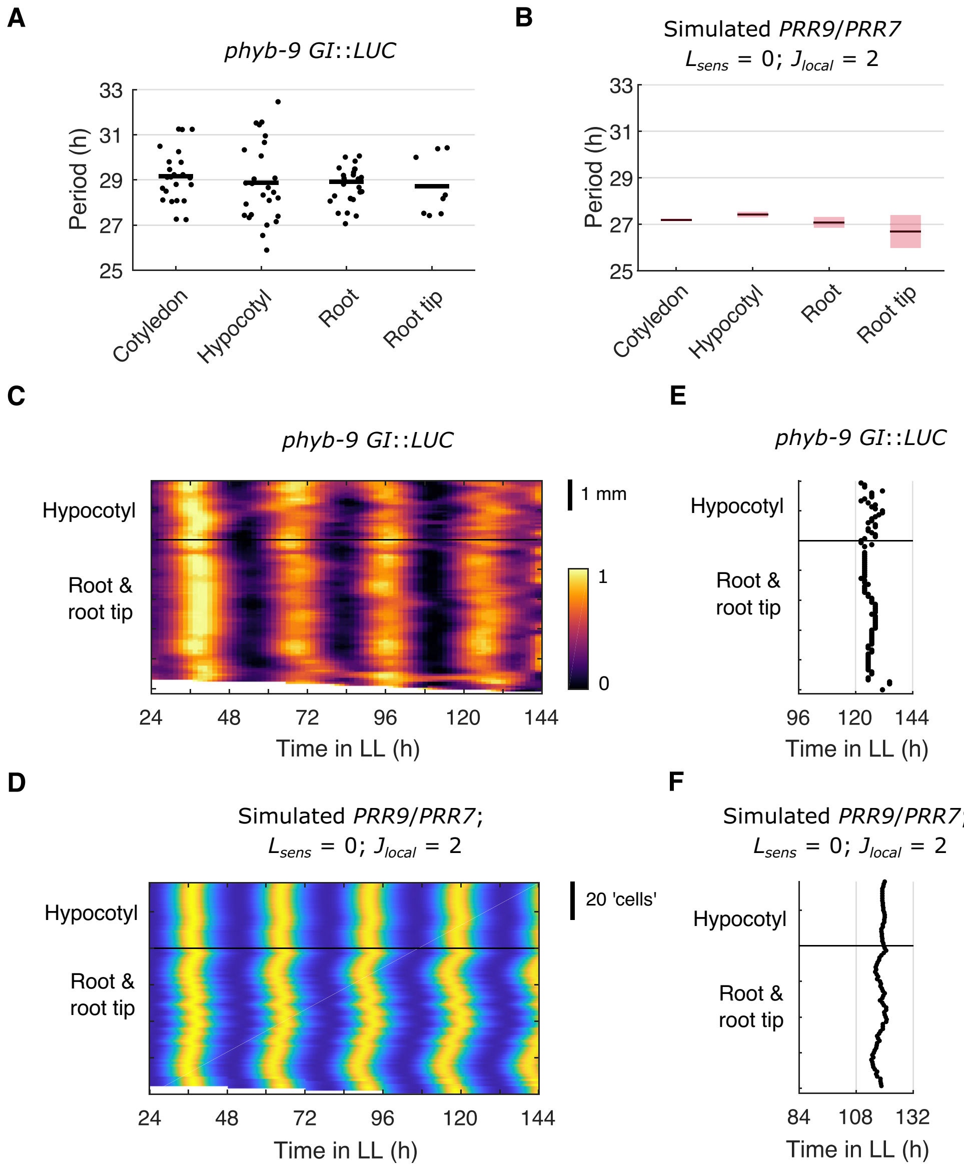

Figure EV1. Simulations with zero light input capture the loss of period differences observed in the *phyb-9* mutant.

A Period estimates of *GI::LUC* expression measured from different organs under red light in the light-sensing mutant *phyb-9* genetic background. Each data point represents a period estimate from the organ of a single seedling and the horizontal black line shows the mean.

B Period estimates of simulated *PRR9/PRR7* expression, measured from regions within the template seedling. Simulations were performed with zero light sensitivity for all cells ($L_{sens} = 0$; $D = 1$) and with local cell-to-cell coupling ($J_{local} = 2$). The horizontal black line shows the mean and the red shaded area one SD of 9 simulations.

C Representative intensity plot of *GI::LUC* expression across longitudinal sections of a single seedling under constant red light. Imaging was performed under red light in the light-sensing mutant *phyb-9* genetic background.

D Representative intensity plot of simulated *PRR9/PRR7* expression across longitudinal sections of a single seedling under LL. Simulations were performed with zero light sensitivity for all cells ($L_{sens} = 0$; $D = 1$) and with local cell-to-cell coupling ($J_{local} = 2$).

E, F Times of the final peaks of *GI::LUC* intensity plot (E) or simulated *PRR9/PRR7* expression (F). Simulations were performed with zero light sensitivity for all cells ($L_{sens} = 0$; $D = 1$) and with local cell-to-cell coupling ($J_{local} = 2$).

Data information: Experimental data is an analysis of *Arabidopsis* time-lapse movies carried out previously (Greenwood *et al*, 2019). $N = 2$ and $n = 8$–23, where N represents the number of independent experiments and n the total number of organs tracked.

 © 2022 The Authors

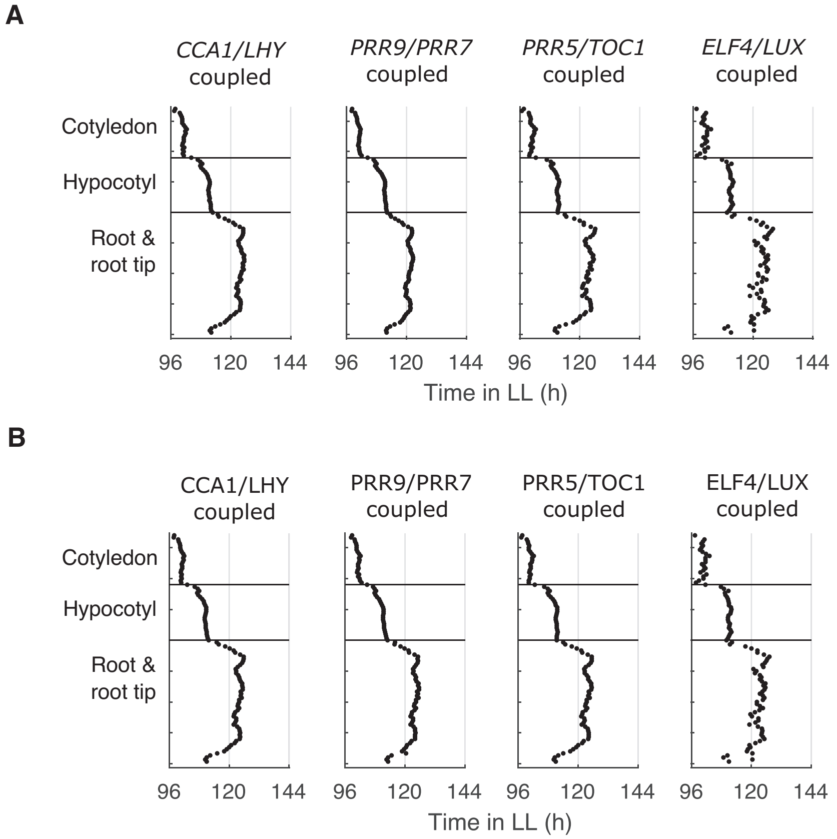

Figure EV2. Peaks of simulated expression with the local sharing of different clock molecules between cells.

A, B The times of the final peaks of simulated *PRR9/PRR7* intensity plots, simulated with the sharing of *CCA1/LHY*, *PRR9/PRR7*, *PRR5/TOC1*, or *ELF4/LUX* mRNA (A) or protein (B) locally between neighbor cells. Simulations were performed under LL with local cell-to-cell coupling ($J_{local} = 2$).

Data information: Data in (A) ("*CCA1/LHY* coupled") is replotted from Fig 3D and data in (B) ("ELF4/LUX coupled") is replotted from Fig 4B.

© 2022 The Authors

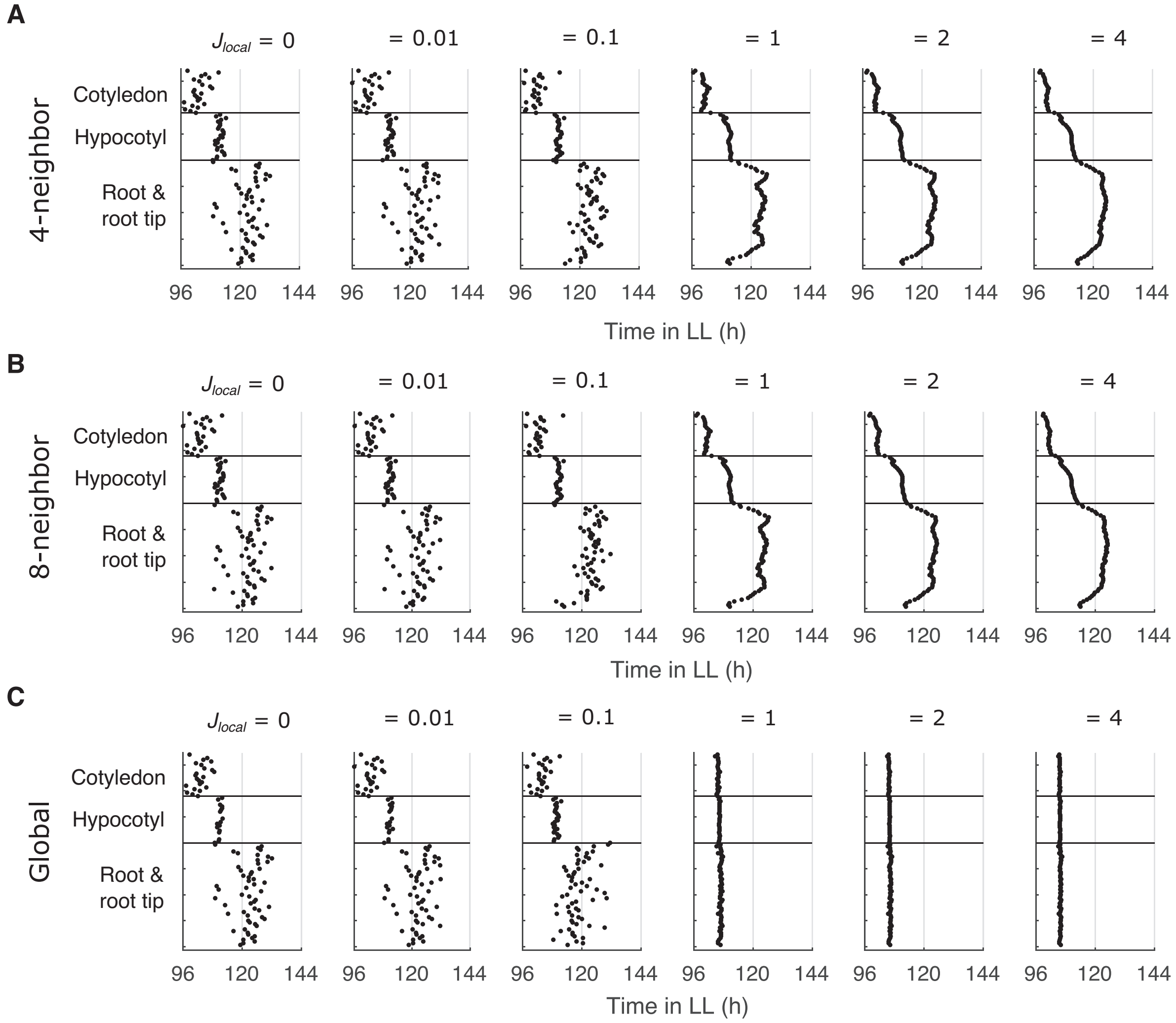

Figure EV3. Peaks of simulated expression under LL with different coupling rules.

A–C The times of the final peaks of simulated *PRR9*/*PRR7* intensity plots, simulated under LL with increasing strengths of local cell-to-cell coupling between the 4 nearest neighbor cells (A), 8 nearest neighbor cells (B), or globally (all-to-all; C).

Data information: (A) is replotted from Fig 3E.

 © 2022 The Authors

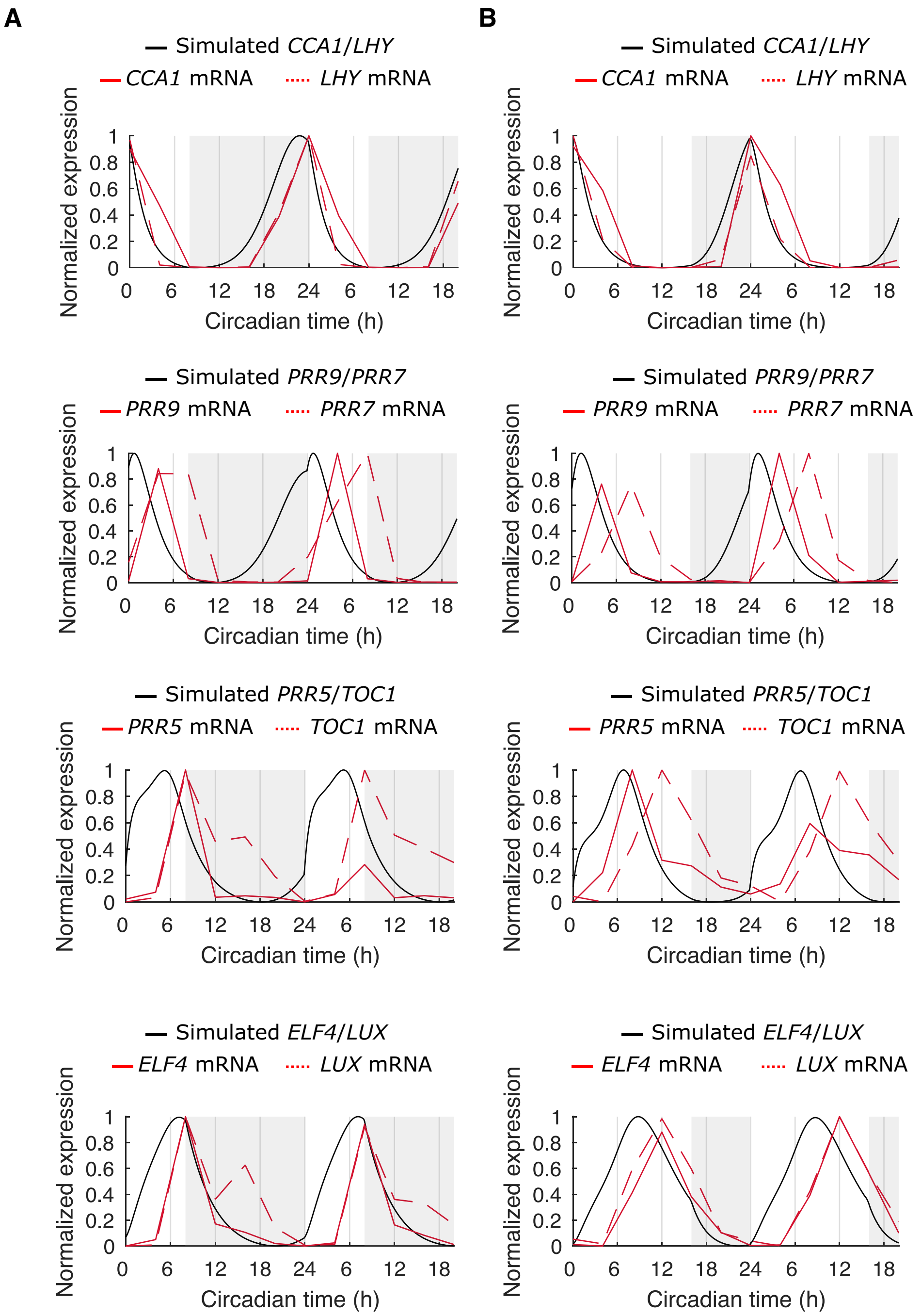

Figure EV4. Simulations under short- and long-day lengths approximate experimental data.

A, B Simulated (black line) and experimental (red line) expression of clock genes under idealized LD cycles with 8 h (A) or 16 h (B) day lengths. Simulations were implemented as a single cell, without variation in gene expression, and with L_{sens} = 1.

Data information: Experimental data are *Arabidopsis* whole-plant averages obtained from the DIURNAL database (Mockler *et al*, 2007).

© 2022 The Authors
