# Supplementary material for: A spatial model of the plant circadian clock reveals design principles for coordinated timing
Source: Mol Syst Biol. 2022 Mar 21;18(3):e10140. doi: 10.15252/msb.202010140 (PMC8935279; doi:10.15252/msb.202010140)
Supplement: Supplementary file 5 [file MSB-18-e10140-s001.pdf]

molecular systems biology

A spatial model of the plant circadian clock reveals design principles for coordinated timing

Mark Greenwood, Isao Tokuda, and James Locke
DOI: 10.15252/msb.202010140

Corresponding author(s): James Locke (james.locke@slcu.cam.ac.uk)

Review Timeline:

Transfer from Review Commons:	23rd Nov 20
Editorial Decision:	25th Nov 20
Revision Received:	24th Nov 21
Editorial Decision:	7th Jan 22
Revision Received:	16th Feb 22
Accepted:	21st Feb 22

Editor: Maria Polychronidou

Transaction Report: This manuscript was transferred to Molecular Systems Biology following peer review at Review Commons.

(Note: With the exception of the correction of typographical or spelling errors that could be a source of ambiguity, letters and reports are not edited. Depending on transfer agreements, referee reports obtained elsewhere may or may not be included in this compilation. Referee reports are anonymous unless the Referee chooses to sign their reports.)

25th Nov 2020

Manuscript Number: MSB-2020-10140, A spatial model of the plant circadian clock reveals design principles for coordinated timing under noisy environments

Dear James,

Thank you for submitting your manuscript to Molecular Systems Biology. I have now read the manuscript and your point-by-point response to the comments of the Review Commons reviewers. I would like to invite you to submit your revised manuscript to Molecular Systems Biology.

Overall, we agree with the reviewers that the presented model seems interesting. The reviewers' concerns seem relatively straightforward to address and I think that your revision plan sounds promising. In particular, it is encouraging to see that you are planning to i) include ELF4 mobility to the model based on the findings of Chen et al, 2020 and ii) perform simulations under short and long days. I think that both these analyses will enhance the impact of the study.

The eventual acceptance of the study will depend on how well the issues raised by the referees have been addressed. As you might already know, our editorial policy allows in principle a single round of major revision, and it therefore is essential to provide responses to the reviewers' comments that are as complete as possible.

To speed up the evaluation of you revised manuscript we would also ask you to address the following editorial points:

- Please provide a .doc version of the manuscript text (including legends for main figures and EV figures) and individual production quality figure files for the main and EV Figures (one file per figure).

- We have replaced Supplementary Information by the Expanded View (EV format). In this case, all additional figures can be included in a PDF called Appendix. Appendix Figures should be labeled and called out as: "Appendix Figure S1, Appendix Figure S2, ... etc.". Each Appendix Figure legend should be provided below the corresponding Figure in the Appendix. Please include a Table of Contents in the beginning of the Appendix. For detailed instructions regarding expanded view please refer to our Author Guidelines: .

- Movies should be provided as Movie EV1, Movie EV2 etc. Please provide each movie in a .zip folder containing a README.txt file with a short description of the movie.

- Due to the quantitative nature of the study we would encourage you to provide the Source Data for the Figure panels showing essential quantitative information. Source Data for main figures should be provided in .zip Folders labeled "Source data for Figure X". Please provide one .zip folder for each of the main figures. Source Data for Appendix Figures should all be provided in one single .zip folder labeled "Source Data for Appendix". Further information regarding Source Data can be found here: .

- Please provide 5 keywords.

- Please include a Conflict of Interest and an Author Contribution statement in the main text.

- Please provide a "standfirst text" summarizing the study in one or two sentences (approximately 250 characters), three to four "bullet points" highlighting the main findings and a "synopsis image" (550px width and max 400px height, jpeg format) to highlight the paper on our homepage.

- All Materials and Methods need to be described in the main text. We would encourage you to use 'Structured Methods', our new Materials and Methods format. According to this format, the Materials and Methods section should include a Reagents and Tools Table (listing key reagents, experimental models, software and relevant equipment and including their sources and relevant identifiers) followed by a Methods and Protocols section in which we encourage the authors to describe their methods using a step-by-step protocol format with bullet points, to facilitate the adoption of the methodologies across labs. More information on how to adhere to this format as well as downloadable templates (.doc or .xls) for the Reagents and Tools Table can be found in our author guidelines: . An example of a Method paper with Structured Methods can be found here:

- Please include a Data availability section describing how the data and code have been made available. This section needs to be formatted according to the example below:
The datasets and computer code produced in this study are available in the following databases:
- Chip-Seq data: Gene Expression Omnibus GSE46748 (https://www.ncbi.nlm.nih.gov/geo/query/acc.cgi?acc=GSE46748)
- Modeling computer scripts: GitHub (https://github.com/SysBioChalmers/GECKO/releases/tag/v1.0)
- [data type]: [full name of the resource] [accession number/identifier] ([doi or URL or identifiers.org/DATABASE:ACCESSION])

- For data quantification: please specify the name of the statistical test used to generate error bars and P values, the number (n) of independent experiments (specify technical or biological replicates) underlying each data point and the test used to calculate p-values in each figure legend. The figure legends should contain a basic description of n, P and the test applied. Graphs must include a description of the bars and the error bars (s.d., s.e.m.).

- When you resubmit your manuscript, please download our CHECKLIST (https://bit.ly/EMBOPressAuthorChecklist) and include the completed form in your submission.
Please note that the Author Checklist will be published alongside the paper as part of the transparent process (https://www.embopress.org/page/journal/17444292/authorguide#transparentprocess).

Please attach a cover letter giving details of the way in which you have handled each of the points raised by the referees. As you probably understand we can give you no guarantee at this stage that the eventual outcome will be favorable.

Feel free to contact me if there is anything you would like to discuss further.

Kind regards,

Maria

Maria Polychronidou, PhD
Senior Editor
Molecular Systems Biology

If you do choose to resubmit, please click on the link below to submit the revision online *within 90 days*.

https://msb.msubmit.net/cgi-bin/main.plex

IMPORTANT: When you send your revision, we will require the following items:
1. the manuscript text in LaTeX, RTF or MS Word format
2. a letter with a detailed description of the changes made in response to the referees. Please specify clearly the exact places in the text (pages and paragraphs) where each change has been made in response to each specific comment given
3. three to four 'bullet points' highlighting the main findings of your study
4. a short 'blurb' text summarizing in two sentences the study (max. 250 characters)
5. a 'thumbnail image' (550px width and max 400px height, Illustrator, PowerPoint or jpeg format), which can be used as 'visual title' for the synopsis section of your paper.
6. Please include an author contributions statement after the Acknowledgements section (see https://www.embopress.org/page/journal/17444292/authorguide)
7. Please complete the CHECKLIST available at (https://bit.ly/EMBOPressAuthorChecklist).
Please note that the Author Checklist will be published alongside the paper as part of the transparent process (https://www.embopress.org/page/journal/17444292/authorguide#transparentprocess).
8. Please note that corresponding authors are required to supply an ORCID ID for their name upon submission of a revised manuscript (EMBO Press signed a joint statement to encourage ORCID adoption).
(https://www.embopress.org/page/journal/17444292/authorguide#editorialprocess)

Currently, our records indicate that the ORCID for your account is 0000-0003-0670-1943.

Please click the link below to modify this ORCID:
Link Not Available

The system will prompt you to fill in your funding and payment information. This will allow Wiley to send you a quote for the article processing charge (APC) in case of acceptance. This quote takes into account any reduction or fee waivers that you may be eligible for. Authors do not need to pay any fees before their manuscript is accepted and transferred to the publisher.

As a matter of course, please make sure that you have correctly followed the instructions for authors as given on the submission website.

*** PLEASE NOTE *** As part of the EMBO Press transparent editorial process initiative (see our Editorial at https://dx.doi.org/10.1038/msb.2010.72), Molecular Systems Biology publishes online a Review Process File with each accepted manuscripts. This file will be published in conjunction with your paper and will include the anonymous referee reports, your point-by-point response and all pertinent correspondence relating to the manuscript. If you do NOT want this File to be

published, please inform the editorial office at msb@embo.org within 14 days upon receipt of the present letter.

***
Rev_Com_number: RC-2020-00464
New_manu_number: MSB-2020-10140
Corr_author: Locke
Title: A spatial model of the plant circadian clock reveals design principles for coordinated timing under noisy environments

Review #1 -

Evidence, reproducibility and clarity (Required)

A. Summary:

In this modeling study, the authors devised a multicellular model to investigate how circadian clocks in different parts (organs) of plants coordinate their timing. The model uses a plausible mechanism to explain how having a different sensitivity to light leads to different phase and period of circadian clock, which is observed in different plant organs. The model allows for entrainment in Light-Dark (LD) cycles and then a release in always-light (LL) environments. The model disentangles numerous factors that have confounded previous experiments. In one instance, the authors assigned different light sensitivities to the different organs (e.g., root tip, hypocotyl, etc.) which unambiguously show that this one element alone - spatially differing sensitivity to light - is sufficient for recapitulating experimentally observed differences in periods and phases between plant organs. The model also recapitulates the spatial waves of gene expression within and between organs that experimentalists reported. At the sub-tissue level, the model-produced waves have similar patterns as the experimentally observed waves. This confirmation further validates the model. By having the cells share clock mRNA, from any clock component genes, showed the same, experimentally observed spatial dynamics. The main conclusion of the study is that regional differences (e.g., between different organs) in light senilities, when combined with cell-to-cell sharing of clock-gene mRNAs, enables a robust, yet flexible, circadian timing under noisy environmental cycles.

B. Specific points:

1.Lines 125-127: "To simulate the variability observed in single cell clock rhythms, we multiplied the level of each mRNA and protein by a time scaling parameter that was randomly selected from a normal distribution." - Why not add a white (Gaussian) noise term to these equations? How does multiplying by a random variable (for rescaling time) different from my proposal? Some explanation should be given in the text here.

2.Does the spatial network model simplify calculations by assuming separations of timescales (e.g., for equilibration in concentrations of mRNAs that diffuse between cells)? If so, it would be good to spell these out in the beginning of the Results section (where the model is described).

3.Lines 161-162: "....in a phase only model by local...." should be "....in a phase model only by local...."

4.Lines 188-190: The authors observed that qualitatively similar/indistinguishable behaviors arose regardless of which elements are varied (e.g., global versus local cell-cell coupling, setting light input to be equal in all regions of the seedling, etc.). Then they claim here that "...these results show that the assumptions of local cell-to-cell coupling and differential light sensitivity between regions are the key aspects of our model that allow a match to experimental data." - I don't see how this follows from the observation almost any of the variations lead to the same behaviors in this section (spatial waves). Show the reasoning in

the text here.

5.Pgs. 9 -10: Section on "Cell-to-cell coupling maintains global coordination under noisy light-dark cycles": The simulation results rigorously support the authors' main conclusion here, which is that local cell-to-cell coupling allows for global coordination under noisy LD cycles. But I'm missing an intuitive explanation (or just any explanation) for why this is. At the end of this section, the authors should provide some intuition or qualitative explanation for the observations that they produced using their model in this section.

6.Lines 261-262: Replace the present tenses with past tenses.

7.Is the main idea that cell-to-cell coupling allows for averaging of fluctuations, between organs or cells within the same organ, while allowing for coordination of the average quantities? Is this responsible for both the flexibility and robustness observed under noisy environmental cycles?

8.Line 304: Is it really true that the mammalian circadian rhythm is centralized? Don't some parts of our bodies have different circadian clock (e.g., slight differences in phase) than some other parts of our bodies?

3. Significance:

Significance (Required)

Overall assessment:

I enthusiastically recommend this work for publication after the authors address my comments below (please see "Specific points").

The model's main strength is that the authors could vary each ingredient separately - light sensitivity of each cell/organ, which gene's mRNA diffuses between cells, cellular noise, local versus global cell-cell coupling, etc. Afterwards, the authors could determine which of these variations produces which experimentally observed behaviors. Another strength of the model is that it can reproduce not just one, but numerous, experimentally observed behaviors that are important for understanding circadian clocks in plants. Thus, the model is grounded in experimental truth and produces experimentally observed results. Crucially, since the authors could vary every single element in the model independently of the other elements, the authors are able to provide plausible explanations for why the experiments produced the results that they did (experimentally, a number of confounding factors prevented one from pinpointing to which element produced which observation).

Another strength of the model is also extendable, by other researchers to investigate other plant physiologies in the future (e.g., circadian clock's influence on cell division). The authors highlight these future uses in the discussion section. Therefore, I believe that this work will be valuable to plant biologists, non-plant biologists who are interested in circadian clocks, and systems biologists in general.

The manuscript is also well written and relatively easy to follow, even for non-plant biologists like myself.

REFEREE'S CROSS-COMMENTING

Comment on Reviewer #2:

I agree with his/her major criticism #3 (ELF4 long-distance movement). I find this to be a reasonable request. Fulfilling it would increase the paper's impact.

Comment on Reviewer #3:

The reviewer's point (1) asks for a reasonable request.
Regarding his/her point (2): This is also reasonable. I'd recommend his/her suggestion (a). In the end, I'd be interested to see how the authors respond to this (what function they choose to let adjacent cells be subjected to some correlated light-input intensity. I'd be happy with something simple such as < intensity > + noise, where <intensity> is a deterministic term that, for example, decreases exponentially as one moves away from some central cell. Basically, I'd let the authors decide how to implement this and accept their current implementation - no correlation in light-intensity between adjacent cells - as an extreme scenario, as this reviewer points out.

Review #2 -

Evidence, reproducibility and clarity (Required)

Summary:

The manuscript presents an improved model of the circadian clock network that accounts for tissue-specific clock behavior, spatial differences in light sensitivity, and local coupling achieved through intercellular sharing of mRNA. In contrast to whole-plant or "phase-only" models, the authors' approach enables them to address the mechanism behind coupling and how the clock maintains regional synchrony in a noisy environment. Using 34 parameters to describe clock activity and applying the properties mentioned above, the authors demonstrate that their model can recapitulate the spatial waves in circadian gene expression observed and can simulate how the plant maintains local synchrony with regional differences in rhythms under noisy LD cycles. Spatial models that incorporate cell-type-specific sensitivities to environmental inputs and local coupling mechanisms will be most accurate for simulating clock activity under natural environments.

*We have the following **major criticisms** as follows*

1) When assigning light sensitivities in different regions of the plant, the authors assign a higher sensitivity value to the root tip (L=1.03) than they do to the other part of the root (L=0.90). We are curious why the root tip would have higher light sensitivity than the rest of the root. Is this based on experimental data (if so, please cite in this section or methods)? It seems that these L values were assigned simply to make sure they recapitulated the period differences observed in Fig. 2A. Are these values based on PhyB expression in those organs? Or perhaps based on cell density in those locations?

2) In the discussion of the test where they set the "light inputs to be equal" in all regions to simulate the phyb-9 mutant, could the authors please clarify whether that means they set the L light sensitivity value equal in all regions?
a. If they are referring to setting the L value equal to all regions, we suggest that this discussion be moved to the section about different light sensitivities instead of the local sharing of mRNA section.
b. Additionally, is it possible to set the light sensitivity to zero for all parts of the plant? We think this would be more suitable to simulate the phyb-9 mutant phenotype.

3) Based on the recent Chen et al. (2020) paper showing ELF4 long-distance movement, we think it would be of great interest for the authors to model ELF4 protein synthesis/translation as the coupling factor, in addition to the modeling using CCA1/LHY mRNA sharing. We understand you may be saving this analysis for a future modeling paper, but this addition to the paper could increase the impact of this paper.

4) This model is able to simulate circadian rhythms under 12:12 LD cycles, which represents two days of the year-the equinoxes. We are curious if the model can simulate rhythms under short days and long days as well. We understand this analysis may be outside the scope of this paper and may require changing the values of the 34 parameters used but think it could be a useful addition here or in future work.

*And **minor criticisms** as follows*

1) In the first paragraph of the results section, it would be helpful for the authors to reference Table S1 when they mention the 34 parameters used to model oscillator function

2) In the first paragraph of the section titled "Local flexibility persists under idealized and noisy LD cycles", it would be helpful for the authors to reference S12 Fig after the last sentence that starts "However, ELF4/LUX appeared more synchronized..."

3) In the first paragraph of the section titled "Cell-to-cell coupling maintains global communication under noisy light-dark cycles", the authors refer to a "Table 1" but I think they mean to refer to Table S1"

4) In Fig. 1, panel C is described as demonstrating the cell-to-cell coupling through the "level of CCA1/LHY". This phrasing is vague and we think could be improved to the "mRNA level of CCA1/LHY".

3. Significance:

Significance (Required)

This work would be broadly interesting to other researchers studying cell-to-cell signaling and coupling of circadian rhythms in plants and other species where spatial waves of gene expression have been observed (i.e., mice and humans). Additionally, the computational modeling aspect of this work was easily interpretable for someone outside this expertise. Our expertise lies in plant circadian biology.

Review #3 -

Evidence, reproducibility and clarity (Required)

Summary:

The authors start by taking a previously published model of the plant circadian clock and implement five changes: 1) updating the network topology to reflect some recent experimental findings, 2) make a spatial model loosely based on a seedling template 3) introduce coupling between cells based on shared levels of CCA1/LHY 4) randomly rescale time in each cell to induce inter-cell differences in period, 5) include a light sensitivity that depends on the region considered.

For a certain configuration of light sensitivities/intensities, the different periods of oscillations in each seedling region roughly match that of experiments. With a sufficiently high coupling between cells, the system can also generate spatial waves, which are also observed in the experimental system.

With pulsed light inputs the spatial pattern is still produced. The authors then investigate the robustness to environmental noise by generating stochastic light signals and show that the global synchrony, as measured with a synchronisation index, increases with cell-to-cell coupling strength. The paper is overall well-written, and the background and details of the analysis are well presented.

Major comments:

For the first part of paper, the output of the model is certainly the focus. There is virtually no discussion of the inferred parameters and how much confidence the authors have in their values.

My main issue with the paper is about the section with noisy light signals, which is included in the title and is ultimately one of the main themes of the article.

Specifically, on line 224:

"This decrease in cell-to-cell variation revealed an underlying spatial structure (Fig 4D, middle and right, and S13 Fig), comparable to that observed under idealized LD cycles (Fig 4B, middle and right, and S12 Fig)."

Firstly, I don't feel these conclusions match with the data presented. Comparing figure 4D middle and right with figure 4B middle and right shows a clear and pronounced loss in spatial structure. In its current form, this statement has to change, but I believe there are at least two other major issues with this figure:

1) The figure is clearly designed to invite a comparison between the noise-free light cycles on the left with the noisy cycles on the right. However, due to how the noisy light is simulated, the variance of light signal increases AND the average intensity of light decreases by 50%. When comparing the left and the right, we therefore don't know whether the changes are due to differences in the average signal or differences from the stochasticity. I think the authors

should simulate a noisy light signal with the same mean intensity level as the deterministic signal.

.

2) The noise model for the light doesn't seem realistic. On line 484 is says:

"We made the simplifying assumption that each cell is exposed to an independent noisy LD cycle due to their unique positions in the environment. LD cycles were input to the molecular model through the parameter L".

In fact, this could be considered as an incredibly complex signal, because for 800 cells it means drawing 800 random light signals. The implication is that two adjacent cells receive statistically independent light signals. Depending on chance, one cell might receive tropical levels of light while its neighbour experiences a cloudy day. This affects the interpretation and conclusions from figures 4 and 5. I propose two different ways of improving the simulation of the noisy light signal:

a) In one extreme case, all cells receive the same noisy light signal, and the other extreme, they all receive independent signals. You could consider a mixture model of light signals, where each cell receives \lambda L_global(t) + (1-\lambda) L_individual(t), where L_global(t) is a global light signal that is shared by all cells and L_individual(t) is a light signal unique to an individual cell. The mixing parameter \lambda controls how similar the light signal is between cells

b) Clearly the light signal will differ depending on the region, but there will be some spatial correlation. You could also consider methods of simulating light such that neighbouring cells receive correlated signals, although this might be difficult.

Assuming that the problem with the mean signal is corrected, do you expect the average spatial pattern to be the same between figure 4 B and D with no coupling (J=0) (although an increase in the variance between cells)? Perhaps not (owing to nonlinearities in the system), but it would be interesting to comment.

The different periods in the different regions of the seedling are caused by differences in light sensitivity, which the authors claim is justified from refs 12-15. An alternative hypothesis is the that biochemical parameters such as degradation rates are different between regions. This is briefly alluded to in the introduction, but I think it would be interesting to discuss further. What would be the pros and cons of the two different mechanisms?

I understand that the authors used a pre-existing model, but I must say that I find the way that light is incorporated into the model a bit confusing.

On line 345 it says:
"L(t) represents the input light signal (L = 0, lights off; L > 0, lights on) and D(t) denotes a corresponding darkness input signal (D = 1, lights off; D = 0, lights on)."

Surely the only thing that matters biophysically is the number of photons hitting the plant? Could you explain why the model needs to have a separate "darkness signal" compared to just a single light signal?

In the model, the light intensity changes depending on the region. It might make more sense

for interpretability if instead there is an additional light-sensitivity coefficient that depends on the region, because at the moment I'm not sure what units L(t) is supposed to take.

Minor comments

Could you more explicitly describe a possible molecular mechanism through which the coupling acts?

In Figure 1C it looks like different genes are coupling to different genes, so you may need to rearrange it.

Line 103: "We found that regional differences persist even under LD cycles, but cell to-cell minimized differences between neighbor cells." Missing word.

Line 124: "The coupling strength was set to 2 (Methods)." This is meaningless in isolation, so it would be better to briefly explain what the coupling parameter is before mentioning its value.

Through the text, I think De Caluwe should be corrected to De Caluwé

Typo line 493

Code and data are not made available.

3. Significance:

Significance (Required)

The authors motivate the paper by highlighting that their proposed model improves on phase-based models in that it describes underlying molecular mechanisms.

From an experimental side, it's interesting that a model is developed and directly compared with measured spatio-temporal waves of gene expression. From a theoretical side, the authors address questions relating to oscillations, multi-scale modelling and noise robustness that also generalise to other systems. I therefore expect that both experimental and theoretical audiences will be interested in the results.

There are many possible additions and modifications that could be made to the model, and so the model and analysis could provide a platform for future research. However, I can't comment on whether there are similar pre-existing models of the plant circadian clock that contain both a molecular description of the circadian clock as well as a spatial scale.

REFEREE'S CROSS-COMMENTING

Comments on Review #1:

The time is rescaled in each cell, meaning that each cell has a unique period, but the dynamics remain deterministic and hence the peak-to-peak times will be exactly the same for each cell. I imagine this isn't completely consistent with single-cell data (if available), where

peak-to-peak times are very likely to be variable due to noisy gene expression. In a future paper it would be interesting to analyse the system using stochastic differential equations.

Comments on Review #2:

I agree on the following two points:

1) It would add value to discuss whether the different ranking of light sensitivities by organ matches any available experimental data.

2) As the Reviewers point out, there are many possibilities for testing the robustness of the system to light clues, including varying the length of the day. Although outside of the scope of this paper, I wonder if it's possible to find data from a light sensor measuring light intensity across an entire year? Plugging such data into the model and measuring how the amplitude and period changes would be really interesting, in my opinion.

We thank the reviewers and editor for their detailed and constructive suggestions for our manuscript. We have carefully implemented their suggestions, including an examination of long-distance coupling and the behavior under different photoperiods. We have also improved the analysis of our model under noisy LD cycles, as proposed by reviewer 3. In addition to these changes, we have made minor improvements to the light inputs in the model (to better match experimental data for PRR5 and TOC1, which are degraded more at night than during the day) and have re-optimized our parameters accordingly. We have altered the title of the manuscript to ‘A spatial model of the plant circadian clock reveals design principles for coordinated timing’, to better describe the breadth of our results. With these changes, we believe the manuscript to be much improved and ready for publication at *Molecular Systems Biology*.

Reviewer #1 (Evidence, reproducibility and clarity):

A. Summary:

In this modeling study, the authors devised a multicellular model to investigate how circadian clocks in different parts (organs) of plants coordinate their timing. The model uses a plausible mechanism to explain how having a different sensitivity to light leads to different phase and period of circadian clock, which is observed in different plant organs. The model allows for entrainment in Light-Dark (LD) cycles and then a release in always-light (LL) environments. The model disentangles numerous factors that have confounded previous experiments. In one instance, the authors assigned different light sensitivities to the different organs (e.g., root tip, hypocotyl, etc.) which unambiguously show that this one element alone - spatially differing sensitivity to light - is sufficient for recapitulating experimentally observed differences in periods and phases between plant organs. The model also recapitulates the spatial waves of gene expression within and between organs that experimentalists reported. At the sub-tissue level, the model-produced waves have similar patterns as the experimentally observed waves. This confirmation further validates the model. By having the cells share clock mRNA, from any clock component genes, showed the same, experimentally observed spatial dynamics. The main conclusion of the study is that regional differences (e.g., between different organs) in light senilities, when combined with cell-to-cell sharing of clock-gene mRNAs, enables a robust, yet flexible, circadian timing under noisy environmental cycles.

Thank you for your review and constructive comments on our work. We have addressed your specific points below.

B. Specific points:

1.Lines 125-127: "To simulate the variability observed in single cell clock rhythms, we multiplied the level of each mRNA and protein by a time scaling parameter that was randomly selected from a normal distribution." - Why not add a white (Gaussian) noise term to these equations? How does multiplying by a random variable (for rescaling time) different from my proposal? Some explanation should be given in the text here.

Thank you for your prompt. We opted for a time scaling approach as this generates between-cell period differences but avoids within-cell period differences. This allows us to

focus on between-cell causes of variation in this paper. We now provide an explanation of this in the text (lines 129-135) and discuss how further work can extend this approach in the discussion (lines 361-370).

2.Does the spatial network model simplify calculations by assuming separations of timescales (e.g., for equilibration in concentrations of mRNAs that diffuse between cells)? If so, it would be good to spell these out in the beginning of the Results section (where the model is described).

We agree with the reviewer that it is important to spell out the assumptions of the model. For computation of the local mean field of the mRNA expressions, we do not consider the time for molecules to diffuse. We have expanded the description of the model at the beginning of the Results section (lines 119-135).

3.Lines 161-162: "....in a phase only model by local...." should be "....in a phase model only by local...."

We referred to models that lack any genetic network information and consider only the phases of individual cellular rhythms as “phase only” models throughout the manuscript. We have now edited this to ‘phase-only’ to avoid any ambiguities such as the one highlighted here by the reviewer.

4.Lines 188-190: The authors observed that qualitatively similar/indistinguishable behaviors arose regardless of which elements are varied (e.g., global versus local cell-cell coupling, setting light input to be equal in all regions of the seedling, etc.). Then they claim here that "...these results show that the assumptions of local cell-to-cell coupling and differential light sensitivity between regions are the key aspects of our model that allow a match to experimental data." - I don't see how this follows from the observation almost any of the variations lead to the same behaviors in this section (spatial waves). Show the reasoning in the text here.

We observed spatial waves with different local coupling regimes (4 and 8 nearest neighbours; Figure EV3A, B). However, we did not observe spatial waves with global coupling (Figure EV3C). This led us to conclude that local coupling is a key aspect. We have also now examined a long-distance coupling regime, and again did not observe spatial waves without local coupling (Figure 4).

In addition, we do not observe waves when setting the light input to be equal in all regions of the seedling (Figure EV1D, F). This confirms that local differences in light sensitivity are also required in our simulations to generate spatial waves. We have now elevated these figures to expanded view format to improve readability, and clarified the points with revisions to the text (lines 196-200).

5.Pgs. 9 -10: Section on "Cell-to-cell coupling maintains global coordination under noisy light-dark cycles": The simulation results rigorously support the authors' main conclusion here, which is that local cell-to-cell coupling allows for global coordination under noisy LD cycles. But I'm missing an intuitive explanation (or just any explanation) for why this is. At the

end of this section, the authors should provide some intuition or qualitative explanation for the observations that they produced using their model in this section.

We have modified our analysis to aid the interpretation and intuition of our results. We introduce the cell timing error (Fig 5C, D) as the difference in peak/trough times of a cell between the idealized and noisy LD condition. Local cell-to-cell coupling decreases the timing error as the reciprocal interactions between cells have a stabilizing effect on the oscillations, increasing their robustness to perturbation by the noisy environment. We have revised the text to provide an intuitive explanation of these results (lines 266-268 and lines 288-292).

6.Lines 261-262: Replace the present tenses with past tenses.

Thank you for your correction. We have fixed this in the text.

7. Is the main idea that cell-to-cell coupling allows for averaging of fluctuations, between organs or cells within the same organ, while allowing for coordination of the average quantities? Is this responsible for both the flexibility and robustness observed under noisy environmental cycles?

The cell-to-cell-coupling allows for the averaging of fluctuations between cells which stabilizes the cellular rhythms, providing robustness to the noisy environment. The between-region phase differences arise from the between-region differences in intrinsic light sensitivities. It was interesting to us that under light-dark cycles the between-region differences persisted despite the stabilizing effect of the coupling. We have revised the text to emphasize these points (lines 288-292). Thank you for your prompts.

8.Line 304: Is it really true that the mammalian circadian rhythm is centralized? Don't some parts of our bodies have different circadian clock (e.g., slight differences in phase) than some other parts of our bodies?

There are indeed some small phase differences between parts of our bodies because the mammalian system, like the plant system, is imperfectly coupled. However, the mammalian system is considered more centralized because the suprachiasmatic nucleus in the brain receives the key entraining signal of light and then coordinates rhythms across the body (Bell-Pedersen et al., 2005, *Nat Rev Gen*; Brown & Azzi, 2013, *Circadian Clocks*). We have added a sentence to clarify this point in the discussion (lines 382-384).

Reviewer #1 (Significance):

Overall assessment:

I enthusiastically recommend this work for publication after the authors address my comments below (please see "Specific points").

The model's main strength is that the authors could vary each ingredient separately - light sensitivity of each cell/organ, which gene's mRNA diffuses between cells, cellular noise, local versus global cell-cell coupling, etc. Afterwards, the authors could determine which of

these variations produces which experimentally observed behaviors. Another strength of the model is that it can reproduce not just one, but numerous, experimentally observed behaviors that are important for understanding circadian clocks in plants. Thus, the model is grounded in experimental truth and produces experimentally observed results. Crucially, since the authors could vary every single element in the model independently of the other elements, the authors are able to provide plausible explanations for why the experiments produced the results that they did (experimentally, a number of confounding factors prevented one from pinpointing to which element produced which observation).

Another strength of the model is also extendable, by other researchers to investigate other plant physiologies in the future (e.g., circadian clock's influence on cell division). The authors highlight these future uses in the discussion section. Therefore, I believe that this work will be valuable to plant biologists, non-plant biologists who are interested in circadian clocks, and systems biologists in general.

The manuscript is also well written and relatively easy to follow, even for non-plant biologists like myself.

Thank you for the positive feedback - we are pleased that you find the manuscript of broad interest to a range of readers. We have updated the paper following your excellent suggestions.

Comment on Reviewer #2:

I agree with his/her major criticism #3 (ELF4 long-distance movement). I find this to be a reasonable request. Fulfilling it would increase the paper's impact.

Please see our response to reviewer #2. We have fulfilled this request and agree that it will increase the papers impact.

Comment on Reviewer #3:

The reviewer's point (1) asks for a reasonable request.
Regarding his/her point (2): This is also reasonable. I'd recommend his/her suggestion (a). In the end, I'd be interested to see how the authors respond to this (what function they choose to let adjacent cells be subjected to some correlated light-input intensity. I'd be happy with something simple such as < intensity > + noise, where is a deterministic term that, for example, decreases exponentially as one moves away from some central cell. Basically, I'd let the authors decide how to implement this and accept their current implementation - no correlation in light-intensity between adjacent cells - as an extreme scenario, as this reviewer points out.

Please see our response to reviewer #3. We have fulfilled this request and now consider multiple scenarios when modeling the environmental noise.

Reviewer #2 (Evidence, reproducibility and clarity):

Summary:

The manuscript presents an improved model of the circadian clock network that accounts for tissue-specific clock behavior, spatial differences in light sensitivity, and local coupling achieved through intercellular sharing of mRNA. In contrast to whole-plant or "phase-only" models, the authors' approach enables them to address the mechanism behind coupling and how the clock maintains regional synchrony in a noisy environment. Using 34 parameters to describe clock activity and applying the properties mentioned above, the authors demonstrate that their model can recapitulate the spatial waves in circadian gene expression observed and can simulate how the plant maintains local synchrony with regional differences in rhythms under noisy LD cycles. Spatial models that incorporate cell-type-specific sensitivities to environmental inputs and local coupling mechanisms will be most accurate for simulating clock activity under natural environments.

Thank you for your review and constructive comments on our work. We have made the following revisions based on your feedback.

*We have the following **major criticisms** as follows*

1) When assigning light sensitivities in different regions of the plant, the authors assign a higher sensitivity value to the root tip (L=1.03) than they do to the other part of the root (L=0.90). We are curious why the root tip would have higher light sensitivity than the rest of the root. Is this based on experimental data (if so, please cite in this section or methods)? It seems that these L values were assigned simply to make sure they recapitulated the period differences observed in Fig. 2A. Are these values based on PhyB expression in those organs? Or perhaps based on cell density in those locations?

We assign the light sensitivity to match observed experimental period differences across the plant (Fig 2A, B). This is based on previous experiments demonstrating that experimental period differences are dependent on light input through the light sensing gene *PHYB* (Greenwood et al., 2019, *PLoS Bio*; Nimmo et al., 2020, *Physiologia Plantarum*). For example, in WT seedlings, the root tip oscillates faster than the root, but this difference is lost in the *phyb-9* mutant (Greenwood et al., 2019). Thus, we assume the root tip to be more sensitive to light than the roots. We have now added a comparison of this experiment to our model (Figure EV1).

Further supporting this assumption, there is evidence that expression of phytochromes and cryptochromes are increased in the root tip relative to the root (e.g., Somers & Quail, 1995, *Plant J*; Bognar et al., 1999, *PNAS*; Toth et al., 2001, *Plant Physiol*), as the reviewer suggests. Further experiments are needed to verify that these transcriptional differences are what lead to the differences in clock timing. We have now added a mention of these papers to the text (lines 148-151).

2) In the discussion of the test where they set the "light inputs to be equal" in all regions to simulate the phyb-9 mutant, could the authors please clarify whether that means they set the L light sensitivity value equal in all regions?

This is indeed what we mean, we have rephrased the text for clarity (lines 163-166).

a. If they are referring to setting the L value equal to all regions, we suggest that this discussion be moved to the section about different light sensitivities instead of the local sharing of mRNA section.

Thank you for your suggestion. We agree and have moved this discussion (lines 163-168).

b. Additionally, is it possible to set the light sensitivity to zero for all parts of the plant? We think this would be more suitable to simulate the phyb-9 mutant phenotype.

We now include simulations with the light sensitivity set to zero in the revised manuscript (Figure EV1). We thank the reviewer for this suggestion.

3) Based on the recent Chen et al. (2020) paper showing ELF4 long-distance movement, we think it would be of great interest for the authors to model ELF4 protein synthesis/translation as the coupling factor, in addition to the modeling using CCA1/LHY mRNA sharing. We understand you may be saving this analysis for a future modeling paper, but this addition to the paper could increase the impact of this paper.

Thank you for the suggestion to improve our manuscript. We agree that it is of interest to model ELF4 protein as the coupling factor. Firstly, in our revision, in addition to each mRNA we now simulate each clock protein as the local coupling factor (Figure EV2).

Secondly, we have now modified the coupling mechanism to simulate the long-distance transport of ELF4 protein proposed by Chen et al., 2020 *Nature Plants*. Our simulations show that alone it cannot drive spatial waves (Figure 4A, B). However, it can create fast periods in the root tip, which when combined with local coupling can drive spatial waves (Figure 4C). We agree with the reviewers that this new result and associated discussion (lines 207-232 and 332-348) will increase the impact of the paper and thank them for their suggestion.

4) This model is able to simulate circadian rhythms under 12:12 LD cycles, which represents two days of the year-the equinoxes. We are curious if the model can simulate rhythms under short days and long days as well. We understand this analysis may be outside the scope of this paper and may require changing the values of the 34 parameters used but think it could be a useful addition here or in future work.

We agree that it is interesting to observe the behavior of the model under different day lengths. We now include single-cell simulations under short and long days, which approximate the phases observed in other groups' whole-plant experimental assays (Figure EV4). In addition, we also now include simulations of our spatial model under short and long days, which predict a spatial structure (Appendix Figure S11). In our revision, we describe these new results in the main text (lines 248-250).

*And **minor criticisms** as follows*

1) In the first paragraph of the results section, it would be helpful for the authors to reference Table S1 when they mention the 34 parameters used to model oscillator function

Thank you, we have now implemented this suggestion.

2) In the first paragraph of the section titled "Local flexibility persists under idealized and noisy LD cycles", it would be helpful for the authors to reference S12 Fig after the last sentence that starts "However, ELF4/LUX appeared more synchronized..."

Thank you, we have now implemented this suggestion (lines 245-248).

3) In the first paragraph of the section titled "Cell-to-cell coupling maintains global communication under noisy light-dark cycles", the authors refer to a "Table 1" but I think they mean to refer to Table S1"

Thank you, we have now implemented this suggestion.

4) In Fig. 1, panel C is described as demonstrating the cell-to-cell coupling through the "level of CCA1/LHY". This phrasing is vague and we think could be improved to the "mRNA level of CCA1/LHY".

Thank you, we have implemented this suggestion.

Reviewer #2 (Significance (Required)):

This work would be broadly interesting to other researchers studying cell-to-cell signaling and coupling of circadian rhythms in plants and other species where spatial waves of gene expression have been observed (i.e., mice and humans). Additionally, the computational modeling aspect of this work was easily interpretable for someone outside this expertise. Our expertise lies in plant circadian biology.

We thank the reviewer for recognising the broad appeal of our work.

Reviewer #3 (Evidence, reproducibility and clarity):

Summary:

The authors start by taking a previously published model of the plant circadian clock and implement five changes: 1) updating the network topology to reflect some recent experimental findings, 2) make a spatial model loosely based on a seedling template 3) introduce coupling between cells based on shared levels of CCA1/LHY 4) randomly rescale time in each cell to induce inter-cell differences in period, 5) include a light sensitivity that depends on the region considered.

For a certain configuration of light sensitivities/intensities, the different periods of oscillations in each seedling region roughly match that of experiments. With a sufficiently high coupling

between cells, the system can also generate spatial waves, which are also observed in the experimental system.

With pulsed light inputs the spatial pattern is still produced. The authors then investigate the robustness to environmental noise by generating stochastic light signals and show that the global synchrony, as measured with a synchronisation index, increases with cell-to-cell coupling strength. The paper is overall well-written, and the background and details of the analysis are well presented.

Thank you for your review and constructive comments on our work. We have made the following revisions based on your specific points.

Major comments:

For the first part of paper, the output of the model is certainly the focus. There is virtually no discussion of the inferred parameters and how much confidence the authors have in their values.

Thank you for this point. We have included a new Appendix Figure examining how the period and amplitudes of the rhythms are affected by a ± 5% change in the parameter values (Appendix Figure 1), and added mention of the inferred parameters to the first section of the Results (lines 115-117).

My main issue with the paper is about the section with noisy light signals, which is included in the title and is ultimately one of the main themes of the article.

Specifically, on line 224:

"This decrease in cell-to-cell variation revealed an underlying spatial structure (Fig 4D, middle and right, and S13 Fig), comparable to that observed under idealized LD cycles (Fig 4B, middle and right, and S12 Fig)."

Firstly, I don't feel these conclusions match with the data presented. Comparing figure 4D middle and right with figure 4B middle and right shows a clear and pronounced loss in spatial structure. In its current form, this statement has to change, but I believe there are at least two other major issues with this figure:

We agree there were some differences in the spatial structure between idealized and noisy conditions in the previous simulations. Further simulations show that this is due to the way we programmed the noisy LD cycles, as the reviewer suggests. We address this further below.

1) The figure is clearly designed to invite a comparison between the noise-free light cycles on the left with the noisy cycles on the right. However, due to how the noisy light is simulated, the variance of light signal increases AND the average intensity of light decreases by 50%. When comparing the left and the right, we therefore don't know whether the changes are due to differences in the average signal or differences from the stochasticity. I

think the authors should simulate a noisy light signal with the same mean intensity level as the deterministic signal.

As mentioned above, we agree that in the previous simulations the average intensity of the light was decreased due to the noise, and this complicated interpretation. We now simulate idealized and noisy light cycles such that the mean light level over the simulations is equal, but retain the day-to-day stochasticity that is observed in the environment (Figure 5A). The spatial structure under idealized (Figure 5B, black dots) and noisy (Figure 5B, red dots) LD condition appears more similar. We thank the reviewer for the helpful suggestion.

2) The noise model for the light doesn't seem realistic. On line 484 is says:

"We made the simplifying assumption that each cell is exposed to an independent noisy LD cycle due to their unique positions in the environment. LD cycles were input to the molecular model through the parameter L".

In fact, this could be considered as an incredibly complex signal, because for 800 cells it means drawing 800 random light signals. The implication is that two adjacent cells receive statistically independent light signals. Depending on chance, one cell might receive tropical levels of light while its neighbour experiences a cloudy day. This affects the interpretation and conclusions from figures 4 and 5. I propose two different ways of improving the simulation of the noisy light signal:

a) In one extreme case, all cells receive the same noisy light signal, and the other extreme, they all receive independent signals. You could consider a mixture model of light signals, where each cell receives \lambda L_global(t) + (1-\lambda) L_individual(t), where L_global(t) is a global light signal that is shared by all cells and L_individual(t) is a light signal unique to an individual cell. The mixing parameter \lambda controls how similar the light signal is between cells

b) Clearly the light signal will differ depending on the region, but there will be some spatial correlation. You could also consider methods of simulating light such that neighbouring cells receive correlated signals, although this might be difficult.

We agree that our current implementation of noisy LD cycles represents an extreme scenario. This scenario may better simulate cellular microenvironments (differences in environment due to a cells position, shading etc.) but poorly simulates weather events. To test the effect of correlations between cells, in our revision we simulate a mixture model of noisy LD cycles, in line with the reviewer’s suggestion (a) (Appendix Figure S13). We observed a qualitatively similar response to coupling with zero and weak correlations. At high correlations, the effect of cell-to-cell coupling was lost. Thus, the stabilizing effect of cell-to-cell coupling depends on some differences in the LD cycle between cells. We describe these results in the main text (lines 284-288) and thank the reviewer for their suggestions.

Assuming that the problem with the mean signal is corrected, do you expect the average spatial pattern to be the same between figure 4 B and D with no coupling (J=0) (although an

increase in the variance between cells)? Perhaps not (owing to nonlinearities in the system), but it would be interesting to comment.

After editing the implementation of the noisy LD signal, the spatial structure under noisy LD (Figure 5B, red dots) is very similar as under idealized LD condition (Figure 5B, black dots). Without local coupling (J_{local} = 0), there is increased variance between cells, however, this variance diminishes with increasing strengths of coupling. We now plot both conditions together to help communicate this result.

The different periods in the different regions of the seedling are caused by differences in light sensitivity, which the authors claim is justified from refs 12-15. An alternative hypothesis is the that biochemical parameters such as degradation rates are different between regions. This is briefly alluded to in the introduction, but I think it would be interesting to discuss further. What would be the pros and cons of the two different mechanisms?

We agree that it is interesting that the oscillators seem to be set by differences in sensitivity to the environment, with differences in biochemical parameters being an alternative mechanism. We have added a paragraph to the discussion speculating on the implications of the different mechanisms (lines 318-330).

I understand that the authors used a pre-existing model, but I must say that I find the way that light is incorporated into the model a bit confusing.

On line 345 it says:
"L(t) represents the input light signal (L = 0, lights off; L > 0, lights on) and D(t) denotes a corresponding darkness input signal (D = 1, lights off; D = 0, lights on)."

Surely the only thing that matters biophysically is the number of photons hitting the plant? Could you explain why the model needs to have a separate "darkness signal" compared to just a single light signal?

A darkness signal has been introduced in many circadian clock models because degradation rates of the clock genes can depend upon the light or dark condition. We have now improved the description of the dark signal (lines 437-440).

In the model, the light intensity changes depending on the region. It might make more sense for interpretability if instead there is an additional light-sensitivity coefficient that depends on the region, because at the moment I'm not sure what units L(t) is supposed to take.

We have now implemented a light sensitivity coefficient, L_{sens}, that depends on the region (described in lines 414-422). We agree that it improves the interpretability and thank the reviewer for the suggestion.

Minor comments

Could you more explicitly describe a possible molecular mechanism through which the coupling acts?

We now explicitly describe the transport mechanisms that we aim to model in each section of the manuscript (lines 127-129 and 208-212). We also expand the discussion to speculate on likely molecules mediating coupling (lines 350-359).

In Figure 1C it looks like different genes are coupling to different genes, so you may need to rearrange it.

We agree that Figure 1C was confusing. We have replaced this figure with a new version, which focuses on the local cell-to-cell coupling scheme. Thank you for pointing this out.

Line 103: "We found that regional differences persist even under LD cycles, but cell to-cell minimized differences between neighbor cells." Missing word.

Thank you, we have now corrected this.

Line 124: "The coupling strength was set to 2 (Methods)." This is meaningless in isolation, so it would be better to briefly explain what the coupling parameter is before mentioning its value.

Thank you for your suggestion, we have now described the coupling function in more detail (lines 123-129).

Through the text, I think De Caluwe should be corrected to De Caluwé

Thank you, we have now corrected this.

Typo line 493

Thank you, we have now corrected this.

Code and data are not made available.

Analysis output of experimental data and simulations, as well as the model code is now available from our project GitLab page:
https://gitlab.com/slcu/teamJL/greenwood_tokuda_etal_2021

Reviewer #3 (Significance (Required)):

The authors motivate the paper by highlighting that their proposed model improves on phase-based models in that it describes underlying molecular mechanisms.

From an experimental side, it's interesting that a model is developed and directly compared with measured spatio-temporal waves of gene expression. From a theoretical side, the authors address questions relating to oscillations, multi-scale modelling and noise robustness that also generalise to other systems. I therefore expect that both experimental and theoretical audiences will be interested in the results.

There are many possible additions and modifications that could be made to the model, and so the model and analysis could provide a platform for future research. However, I can't comment on whether there are similar pre-existing models of the plant circadian clock that contain both a molecular description of the circadian clock as well as a spatial scale.

We appreciate the reviewers view that the work is interesting to both experimental and theoretical audiences.

Comments on Review #1:

The time is rescaled in each cell, meaning that each cell has a unique period, but the dynamics remain deterministic and hence the peak-to-peak times will be exactly the same for each cell. I imagine this isn't completely consistent with single-cell data (if available), where peak-to-peak times are very likely to be variable due to noisy gene expression. In a future paper it would be interesting to analyse the system using stochastic differential equations.

Please see our response to reviewer #1. We have fulfilled this request by improved discussion of our approach and potential future directions.

Comments on Review #2:

I agree on the following two points:

1) It would add value to discuss whether the different ranking of light sensitivities by organ matches any available experimental data.

Please see our response to reviewer #2. We have fulfilled this request by discussion of the relevant experiments, and comparison of our model to some of the experimental data.

2) As the Reviewers point out, there are many possibilities for testing the robustness of the system to light clues, including varying the length of the day. Although outside of the scope of this paper, I wonder if it's possible to find data from a light sensor measuring light intensity across an entire year? Plugging such data into the model and measuring how the amplitude and period changes would be really interesting, in my opinion.

Thank you for your suggestion. We also see this as an exciting future direction.

7th Jan 2022

Manuscript Number: MSB-2020-10140R
Title: A spatial model of the plant circadian clock reveals design principles for coordinated timing

Dear James,

Thank you again for submitting your revised study to Molecular Systems Biology along with the referee reports from Review Commons. We have now heard back from the three reviewers who were asked to evaluate your revised study. As you will see below, the reviewers are satisfied with the performed revisions and they are supportive of publication in Molecular Systems Biology. Reviewer #3 only lists a rather minor concern, which can be addressed in a minor revision.

We would also ask you to address some remaining editorial issues listed below:

- On page 11 please correct "Appendix Table 1" to "Appendix Table S1".

- Our data integrity analyst noted a few instances of figure panel reuse i.e. Figure 2E in Figure S8A, Figure EV3A in Figure S8A, and Figure S10A in Figure S11A. We would ask you to indicate the data/panel reuse in the respective figure legends for transparency.

- Please format the reference list according to the MSB style i.e. listing the first 10 authors followed by et al. The references should be sorted in alphabetical order.

- The labelling in the synopsis image does not read very well at the required final size (width = 550 px). Please provide an updated synopsis (.jpg or .png file) exactly at 550 px width (the height does not matter), ensuring that all labelling is easily readable and that the resolution is adequate.

Please resubmit your revised manuscript online, with a covering letter listing amendments and responses to each point raised by the referees. Please resubmit the paper **within one month** and ideally as soon as possible. If we do not receive the revised manuscript within this time period, the file might be closed and any subsequent resubmission would be treated as a new manuscript. Please use the Manuscript Number (above) in all correspondence.

Click on the link below to submit your revised paper.

Link Not Available

Thank you for submitting this paper to Molecular Systems Biology.

Best wishes and Happy New Year,

Maria

Maria Polychronidou, PhD
Senior Editor
Molecular Systems Biology

If you do choose to resubmit, please click on the link below to submit the revision online before 6th Feb 2022.

Link Not Available

*** PLEASE NOTE *** As part of the EMBO Publications transparent editorial process initiative (see our Editorial at https://www.nature.com/msb/journal/v6/n1/full/msb201072.html), Molecular Systems Biology will publish online a Review Process File to accompany accepted manuscripts. When preparing your letter of response, please be aware that in the event of acceptance, your cover letter/point-by-point document will be included as part of this File, which will be available to the scientific community. More information about this initiative is available in our Instructions to Authors. If you have any questions about this initiative, please contact the editorial office (msb@embo.org).

Reviewer #1:

I recommend publication without further revisions.

The authors have done an excellent job of revising the manuscript and addressing most of my and the other reviewers' comments.

I particularly like the additional work that examined long-distance coupling regime in which tehy did not observe spatial waves without local coupling (fig. 4). This strengthens their main conclusion.

Congratulations on this excellent paper.

Reviewer #2:

The authors satisfactorily addressed our concerns through new analysis, additional text, figures, and editing. We find the new manuscript is increased in clarity and broadened in impact. We appreciate the thoughtful rebuttal and revision.

Reviewer #3:

The authors have provided an extensively revised manuscript that systematically adresses the reviewer comments from the first revision.

In particular, the new Figure S13 makes the effect of the noise in the light source more transparent, and the responses to all my comments from the first version are satisfactory.

Perhaps my only remaining reservations from a methodological perspective would be the parameter uncertainty quantification. The authors have supplied a new Figure S1 where all model parameters were uniformly distributed in the {plus minus} 5 % range of the optimal values and the free running periods and amplitudes were calculated. We at least see that there are no dramatic changes in the model output as the parameters are varied, but it remains somewhat unsatifactory in the sense that we don't see confidence intervals for parameter estimates. There are some methods from e.g. Approximate Bayesian Computation that might be useful here, but the inference scheme would probably be complicated. Perhaps a short comment about this in the Discussion might be useful.

Congratulations to the authors for an interesting article.

***

Rev_Com_number: RC-2020-00464
New_manu_number: MSB-2020-10140R
Corr_author: Locke
Title: A spatial model of the plant circadian clock reveals design principles for coordinated timing

We thank the three reviewers for their positive assessment of our paper. We note the one request from Reviewer 2:

‘Perhaps my only remaining reservations from a methodological perspective would be the parameter uncertainty quantification. The authors have supplied a new Figure S1 where all model parameters were uniformly distributed in the {plus minus} 5 % range of the optimal values and the free running periods and amplitudes were calculated. We at least see that there are no dramatic changes in the model output as the parameters are varied, but it remains somewhat unsatifactory in the sense that we don't see confidence intervals for parameter estimates. There are some methods from e.g. Approximate Bayesian Computation that might be useful here, but the inference scheme would probably be complicated. Perhaps a short comment about this in the Discussion might be useful.’

To address this concern, we have added the following text in the description of the parameter sensitivity analysis method (lines 514-516):

‘Our sensitivity analysis could in future be extended (e.g. by using Approximate Bayesian Computation) to calculate confidence intervals for the parameter estimates.’

In addition to fulfilling this request, we have made two minor changes to the figures, as outlined below,

1. We have changed Figure EV1B so that it plots the periods for *PRR9*/*PRR7* expression instead of *PRR5*/*TOC1*. We make this change for clarity as we plot *PRR9*/*PRR7* simulations in the remainder of the figure.
2. We have fixed a plotting mistake in Appendix Figure S6B and S6D, so that it now shows the simulation repeat that matches the peak times displayed in Appendix Figure S6F and S6H.

21st Feb 2022

Manuscript number: MSB-2020-10140RR, A spatial model of the plant circadian clock reveals design principles for coordinated timing

Dear James,

Thank you again for sending us your revised manuscript. We are now satisfied with the modifications made and I am pleased to inform you that your paper has been accepted for publication.

*** PLEASE NOTE *** As part of the EMBO Publications transparent editorial process initiative (see our Editorial at https://dx.doi.org/10.1038/msb.2010.72), Molecular Systems Biology publishes online a Review Process File with each accepted manuscripts. This file will be published in conjunction with your paper and will include the anonymous referee reports, your point- by-point response and all pertinent correspondence relating to the manuscript. If you do NOT want this File to be published, please inform the editorial office at msb@embo.org within 14 days upon receipt of the present letter.

Should you be planning a Press Release on your article, please get in contact with msb@wiley.com as early as possible, in order to coordinate publication and release dates.

LICENSE AND PAYMENT:
All articles published in Molecular Systems Biology are fully open access: immediately and freely available to read, download and share.

Molecular Systems Biology charges an article processing charge (APC) to cover the publication costs. You, as the corresponding author for this manuscript, should have already received a quote with the article processing fee separately. Please let us know in case this quote has not been received.

Once your article is at Wiley for editorial production you will receive an email from Wiley's Author Services system, which will ask you to log in and will present you with the publication license form for completion. Within the same system the publication fee can be paid by credit card, an invoice or pro forma can be requested.

Payment of the publication charge and the signed Open Access Agreement form must be received before the article can be published online.

Molecular Systems Biology articles are published under the Creative Commons licence CC BY, which facilitates the sharing of scientific information by reducing legal barriers, while mandating attribution of the source in accordance to standard scholarly practice.

Proofs will be forwarded to you within the next 2-3 weeks.

Thank you very much for submitting your work to Molecular Systems Biology.

Kind regards,

Maria

Maria Polychronidou, PhD
Senior Editor
Molecular Systems Biology

***
Rev_Com_number: RC-2020-00464
New_manu_number: MSB-2020-10140RR
Corr_author: Locke
Title: A spatial model of the plant circadian clock reveals design principles for coordinated timing

EMBO PRESS

YOU MUST COMPLETE ALL CELLS WITH A PINK BACKGROUND

PLEASE NOTE THAT THIS CHECKLIST WILL BE PUBLISHED ALONGSIDE YOUR PAPER

Corresponding Author Name: James Locke	
Journal Submitted to: Molecular Systems Biology	
Manuscript Number: MSB-2020-10140	

Reporting Checklist For Life Sciences Articles (Rev. June 2017)

This checklist is used to ensure good reporting standards and to improve the reproducibility of published results. These guidelines are consistent with the Principles and Guidelines for Reporting Preclinical Research issued by the NIH in 2014. Please follow the journal's authorship guidelines in preparing your manuscript.

A- Figures

1. Data

The data shown in figures should satisfy the following conditions:

- the data were obtained and processed according to the field's best practice and are presented to reflect the results of the experiments in an accurate and unbiased manner.
- figure panels include only data points, measurements or observations that can be compared to each other in a scientifically meaningful way.
- graphs include clearly labeled error bars for independent experiments and sample sizes. Unless justified, error bars should not be shown for technical replicates.
- if n< 5, the individual data points from each experiment should be plotted and any statistical test employed should be justified
- Source Data should be included to report the data underlying graphs. Please follow the guidelines set out in the author ship guidelines on Data Presentation.

2. Captions

Each figure caption should contain the following information, for each panel where they are relevant:

- a specification of the experimental system investigated (eg cell line, species name).
- the assay(s) and method(s) used to carry out the reported observations and measurements
- an explicit mention of the biological and chemical entity(ies) that are being measured.
- an explicit mention of the biological and chemical entity(ies) that are altered/varied/perturbed in a controlled manner.

- the exact sample size (n) for each experimental group/condition, given as a number, not a range;
- a description of the sample collection allowing the reader to understand whether the samples represent technical or biological replicates (including how many animals, litters, cultures, etc.).
- a statement of how many times the experiment shown was independently replicated in the laboratory.
- definitions of statistical methods and measures:
  - common tests, such as t-test (please specify whether paired vs. unpaired), simple χ2 tests, Wilcoxon and Mann-Whitney tests, can be unambiguously identified by name only, but more complex techniques should be described in the methods section;
  - are tests one-sided or two-sided?
  - are there adjustments for multiple comparisons?
  - exact statistical test results, e.g., P values = x but not P values < x;
  - definition of 'center values' as median or average;
  - definition of error bars as s.d. or s.e.m.

Any descriptions too long for the figure legend should be included in the methods section and/or with the source data.

In the pink boxes below, please ensure that the answers to the following questions are reported in the manuscript itself. Every question should be answered. If the question is not relevant to your research, please write NA (non applicable). We encourage you to include a specific subsection in the methods section for statistics, reagents, animal models and human subjects.

USEFUL LINKS FOR COMPLETING THIS FORM

http://www.antibodypedia.com
http://1degreebio.org
http://www.equator-network.org/reporting-guidelines/improving-bioscience-research-repor
http://grants.nih.gov/grants/olaw/olaw.htm
http://www.mrc.ac.uk/Ourresearch/Ethicsresearchguidance/Useofanimals/index.htm
http://ClinicalTrials.gov
http://www.consort-statement.org
http://www.consort-statement.org/checklists/view/32-consort/66-title
http://www.equator-network.org/reporting-guidelines/reporting-recommendations-for-tum
http://datadryad.org
http://figshare.com
http://www.ncbi.nlm.nih.gov/gap
http://www.ebi.ac.uk/ega
http://biomodels.net/
http://biomodels.net/miriam/
http://jjj.biochem.sun.ac.za
http://oba.od.nih.gov/biosecurity/biosecurity_documents.html
http://www.selectagents.gov/

B- Statistics and general methods

Please fill out these boxes (Do not worry if you cannot see all your text once you press return)

1.a. How was the sample size chosen to ensure adequate power to detect a pre-specified effect size?	No statistical tests were used to select the sample size.
1.b. For animal studies, include a statement about sample size estimate even if no statistical methods were used.	NA
2. Describe inclusion/exclusion criteria if samples or animals were excluded from the analysis. Were the criteria pre-established?	Seedlings were excluded from the analysis if they were not rhythmic, as described in the previous experimental studies (Greenwood et al., 2019 PLoS Biology; Gould et al., 2018 eLife; Mockler et al., 2007 Cold Spring Harbor Symposia on Quantitative Biology).
3. Were any steps taken to minimize the effects of subjective bias when allocating animals/samples to treatment (e.g. randomization procedure)? If yes, please describe.	NA
For animal studies, include a statement about randomization even if no randomization was used.	NA
4.a. Were any steps taken to minimize the effects of subjective bias during group allocation or/and when assessing results (e.g. blinding of the investigator)? If yes please describe.	None.
4.b. For animal studies, include a statement about blinding even if no blinding was done	NA
5. For every figure, are statistical tests justified as appropriate?	NA
Do the data meet the assumptions of the tests (e.g., normal distribution)? Describe any methods used to assess it.	NA
Is there an estimate of variation within each group of data?	All summary statistics include an estimate of variation.

Is the variance similar between the groups that are being statistically compared?	NA

C- Reagents

6. To show that antibodies were profiled for use in the system under study (assay and species), provide a citation, catalog number and/or clone number, supplementary information or reference to an antibody validation profile. e.g., Antibodypedia (see link list at top right), 1DegreeBio (see link list at top right).	NA
7. Identify the source of cell lines and report if they were recently authenticated (e.g., by STR profiling) and tested for mycoplasma contamination.	NA

* for all hyperlinks, please see the table at the top right of the document

D- Animal Models

8. Report species, strain, gender, age of animals and genetic modification status where applicable. Please detail housing and husbandry conditions and the source of animals.	NA
9. For experiments involving live vertebrates, include a statement of compliance with ethical regulations and identify the committee(s) approving the experiments.	NA
10. We recommend consulting the ARRIVE guidelines (see link list at top right) (PLoS Biol. 8(6), e1000412, 2010) to ensure that other relevant aspects of animal studies are adequately reported. See author guidelines, under 'Reporting Guidelines'. See also: NIH (see link list at top right) and MRC (see link list at top right) recommendations. Please confirm compliance.	NA

E- Human Subjects

11. Identify the committee(s) approving the study protocol.	NA
12. Include a statement confirming that informed consent was obtained from all subjects and that the experiments conformed to the principles set out in the WMA Declaration of Helsinki and the Department of Health and Human Services Belmont Report.	NA
13. For publication of patient photos, include a statement confirming that consent to publish was obtained.	NA
14. Report any restrictions on the availability (and/or on the use) of human data or samples.	NA
15. Report the clinical trial registration number (at ClinicalTrials.gov or equivalent), where applicable.	NA
16. For phase II and III randomized controlled trials, please refer to the CONSORT flow diagram (see link list at top right) and submit the CONSORT checklist (see link list at top right) with your submission. See author guidelines, under 'Reporting Guidelines'. Please confirm you have submitted this list.	NA
17. For tumor marker prognostic studies, we recommend that you follow the REMARK reporting guidelines (see link list at top right). See author guidelines, under 'Reporting Guidelines'. Please confirm you have followed these guidelines.	NA

F- Data Accessibility

18: Provide a "Data Availability" section at the end of the Materials & Methods, listing the accession codes for data generated in this study and deposited in a public database (e.g. RNA-Seq data: Gene Expression Omnibus GSE39462, Proteomics data: PRIDE PXD000208 etc.) Please refer to our author guidelines for 'Data Deposition'.<br><br>Data deposition in a public repository is mandatory for:<br>a. Protein, DNA and RNA sequences<br>b. Macromolecular structures<br>c. Crystallographic data for small molecules<br>d. Functional genomics data<br>e. Proteomics and molecular interactions	A "Data Availability" section is included at the end of the Materials & Methods.
19. Deposition is strongly recommended for any datasets that are central and integral to the study; please consider the journal's data policy. If no structured public repository exists for a given data type, we encourage the provision of datasets in the manuscript as a Supplementary Document (see author guidelines under 'Expanded View' or in unstructured repositories such as Dryad (see link list at top right) or Figshare (see link list at top right).	Analysis output of experimental data and simulations is available from our project GitLab page.
20. Access to human clinical and genomic datasets should be provided with as few restrictions as possible while respecting ethical obligations to the patients and relevant medical and legal issues. If practically possible and compatible with the individual consent agreement used in the study, such data should be deposited in one of the major public access-controlled repositories such as dbGAP (see link list at top right) or EGA (see link list at top right).	NA
21. Computational models that are central and integral to a study should be shared without restrictions and provided in a machine-readable form. The relevant accession numbers or links should be provided. When possible, standardized format (SBML, CellML) should be used instead of scripts (e.g. MATLAB). Authors are strongly encouraged to follow the MIRIAM guidelines (see link list at top right) and deposit their model in a public database such as Biomodels (see link list at top right) or JWS Online (see link list at top right). If computer source code is provided with the paper, it should be deposited in a public repository or included in supplementary information.	Computer model code is available from our public GitLab repository.

G- Dual use research of concern

22. Could your study fall under dual use research restrictions? Please check biosecurity documents (see link list at top right) and list of select agents and toxins (APHIS/CDC) (see link list at top right). According to our biosecurity guidelines, provide a statement only if it could.	NA
